# Integrating ethics in AI development: a qualitative study

**DOI:** 10.1186/s12910-023-01000-0

**Published:** 2024-01-23

**Authors:** Laura Arbelaez Ossa, Giorgia Lorenzini, Stephen R. Milford, David Shaw, Bernice S. Elger, Michael Rost

**Affiliations:** 1 Institute for Biomedical Ethics, University of Basel, Basel, Switzerland; 2 Care and Public Health Research Institute, Maastricht University, Maastricht, Netherlands; 3 Center for Legal Medicine (CURML), University of Geneva, Geneva, Switzerland

**Keywords:** Artificial intelligence, AI, Qualitative research, AI ethics, AI development, AI guidance, Implementation

## Abstract

**Background:**

While the theoretical benefits and harms of Artificial Intelligence (AI) have been widely discussed in academic literature, empirical evidence remains elusive regarding the practical ethical challenges of developing AI for healthcare. Bridging the gap between theory and practice is an essential step in understanding how to ethically align AI for healthcare. Therefore, this research examines the concerns and challenges perceived by experts in developing ethical AI that addresses the healthcare context and needs.

**Methods:**

We conducted semi-structured interviews with 41 AI experts and analyzed the data using reflective thematic analysis.

**Results:**

We developed three themes that expressed the considerations perceived by experts as essential for ensuring AI aligns with ethical practices within healthcare. The first theme explores the ethical significance of introducing AI with a clear and purposeful objective. The second theme focuses on how experts are concerned about the tension that exists between economic incentives and the importance of prioritizing the interests of doctors and patients. The third theme illustrates the need to develop context-sensitive AI for healthcare that is informed by its underlying theoretical foundations.

**Conclusions:**

The three themes collectively emphasized that beyond being innovative, AI must genuinely benefit healthcare and its stakeholders, meaning AI also aligns with intricate and context-specific healthcare practices. Our findings signal that instead of narrow product-specific AI guidance, ethical AI development may need a systemic, proactive perspective that includes the ethical considerations (objectives, actors, and context) and focuses on healthcare applications. Ethically developing AI involves a complex interplay between AI, ethics, healthcare, and multiple stakeholders.

**Supplementary Information:**

The online version contains supplementary material available at 10.1186/s12910-023-01000-0.

## Introduction

The application of Artificial Intelligence (AI) in medicine has become a focus of academic discussions, given its (potentially) disruptive effects on healthcare processes, expectations, and relationships. While many see AI's potential to utilize vast data to improve healthcare and support better clinical decisions, there are also increasing concerns and challenges in aligning AI with ethical practices [1]. To set the right process and ethical goals, governmental and private institutions have developed many recommendations to guide the development of AI [2, 3]. These documents have common themes of designing AI to ensure it is robust, safe, fair, and trustworthy using complementary bioethical principles [4]. Although these recommendations have served as building blocks of a common ethical framework for AI, less guidance exists on how these ideals must be translated into practical considerations [3–6]. Beyond ethical considerations, there is also an ethical imperative that AI complies practically with minimal performance, testing, or therapeutic value requirements as with other medical care products [7].

While many ethical considerations may not be unique to AI—such as respecting patients’ autonomy or ensuring healthcare remains fair for all—modern AI techniques (especially in opaque Machine Learning (ML) programs) present more challenges to ensure ethical compliance. In comparison with traditional expert systems that rely on visible and understandable sets of if-not statements, many ML techniques have multiple data connections that are less conducive to direct and fruitful human oversight because of the inherent complexity of these systems or many techniques lacking transparency due to hidden decision layers [1]. In that sense, ensuring AI systems fulfill healthcare’s ethical ideals cannot rely solely on oversight or supervision of their behavior. Ethical ideals and concepts must be often embedded in all steps of AI’s lifecycle, from ideation to development and implementation. Epley and Tannenbaum wrote in “Treating Ethics as a Design Problem” that to develop interventions and policies that encourage ethical AI, the focus must be on the process and context of its development, making ethical considerations part of the practicalities of day-to-day routines to an extent that they should become ingrained habits in practice [8].

In revising common tools to translate AI ethics into practice, Morley et al. found that most tools focus on documenting decision-making rather than guiding how to design ethical AI (to make it more or less ethically aligned) [4]. In that sense, these tools offer less support in understanding how to achieve ethical AI in practice or “in the wild”. Two common frameworks for the development of AI are ethics-by-design and responsible research and innovation (RRI), both aim to promote that those developing AI consider ethical aspects or how to make AI ethically acceptable, sustainable, and socially desirable [9–11]. While both frameworks focus on ethically developing AI, they highlight questions or potential ethical concerns rather than actionable steps. Therefore, challenges remain in translating theoretical discussions into practical impacts [3, 12–14].

Evidence from previous qualitative research with participants working in the technology industry demonstrated that the gap between ethical theory and practice exists to a greater degree than initially considered [15–17]. There is a recognition that ethical practices must be defined by sector, application, and project type, as widespread generic guidance may not answer to context-specific complexities [16, 17]. This is not the current development trend, as most AI ethics guides are generic and non-sector-specific [2, 3]. Indeed, specific ethical guidelines for AI in healthcare, are scarce, and even more so when particular AI applications are considered. Given the lack of practical recommendations, researchers found that most companies first develop AI systems and only then attempt to understand how to generate ethical principles and standardize best practices, instead of integrating ethical considerations into their daily operations [17]. In a way, most ethical recommendations for AI’s development may have a “post” orientation where ethical values and consequences are considered “*afterward and just adapted to actions, but do not align action accordingly*” [18]. For example, researchers found that software developers and engineering students did not change their established ways of working even when a code of ethics was widely available and recommended [15].

Rising to the challenge of designing and deploying ethical AI to serve healthcare is essential. Still, many questions remain regarding the characteristics and processes that would support AI's ethical development and implementation. Most researchers have focused on “consumer research” on the conditions for people to accept the usage of AI [19]. In two recently published systematic reviews of empirical research available for AI in healthcare, most studies explored the knowledge and attitudes towards AI or factors contributing to stakeholders’ acceptance or adoption [20, 21]. However, how AI is developed may affect its acceptance by stakeholders or usage. According to the systematic review by Tang et al., only 1.8% of all empirical evidence focused on AI’s ethical issues, which signaled the existing gap between ethical aspects of AI development and connecting high-level ethical principles to practices [21]. Given that evidence is limited regarding the integration of ethics into AI’s development, this research examines the challenges experts perceive in developing ethical AI for healthcare. However, the focus is not on theoretically discussing the ethical risk of AI in healthcare, nor ethical principles, but on how practical aspects may also benefit from being ethically approached during the development and implementation of AI in healthcare. As such, this research is a step forward in bridging the gap between ethical theory and practice for healthcare AI. As acknowledged by Mittelstadt, “ethics is a process, not a destination,” and the real work of AI ethics research must focus on translating and implementing principles to practice not in a formulaic way but as a path to understanding the real ethical challenges of AI that may go unnoticed in theoretical discussions [5]. Therefore, we used a qualitative approach to explore the topic from the views of professionals involved in developing and using AI (hereafter: AI experts). This paper aims to provide insights to identify the practical ethical challenges of developing AI for healthcare. Our contribution aims to obtain empirical evidence and contribute to the debate on potential practical challenges that may go unnoticed in theoretical ethical discussions, especially when AI is used in Clinical Decision Support Systems (CDSS).

## Methods

The results presented in this paper are part of a research project funded by the Swiss National Research Program, “EXPLaiN”, which critically evaluates “Ethical and Legal Issues of Mobile Health-Data”. For reporting the methods and results, we followed the criteria for qualitative research (SRQR) [22]. All experimental protocols were approved by the Ethics Committee of Northwestern and Central Switzerland (EKNZ). This project was done under the regulatory framework for Human Research ACT in Switzerland. After revision, the EKNZ issued a waiver statement (declaration of no objection: AO_2021-00045) declaring that informed consent was not needed for experts. However, informed consent was verbally obtained from all subjects and audio-recorded at the beginning of each interview.

### Participants recruitment

To be eligible for recruitment, AI experts had to have experience working with or developing AI for healthcare, allowing us to explore the views of various professional backgrounds. Given that AI for healthcare is a multi- and interdisciplinary field, exploring multiple backgrounds provided insights into AI ethical practices beyond professional silos. We utilized professional networks and contacted authors of academic publications in AI. Using purposive sampling based on experience and exposure to AI allowed us to produce rich, articulated, and expressive data [23].

### Data collection

We used semi-structured interview guides to allow for this study's exploratory approach. An interview guide was developed by the research team and included questions regarding the utilization of AI in healthcare, focusing on key domains: (i) overall perceptions of AI, (ii) AI as a direct-to-patient solution (in the form of wearables), (iii) the dynamics of AI within doctor-patient interactions. After piloting the interview guide with six participants, we decided to contextualize the questions using vignettes (a situation description) to probe for an in-depth discussion. The vignettes were highly plausible scenarios of current and future AI interactions with patients (via smartwatches) or doctors via a CDSS. Vignettes probe for attitudes and beliefs while focusing less on the theoretical knowledge within the research area [24, 25]. Although we recognize that vignette responses are primarily based on personal views and moral intuitions rather than being theoretically grounded, how participants interpret the vignette is similar to how they make sense of a situation and make decisions [24]. The guideline for the semi-structured interview is available in the Supplementary materials.

Two research team members conducted the interviews (L.A.O *n* = 21; G.L. *n* = 20) between October 2021 and April 2022. All interviews were held in English and audio-recorded using Zoom but stored locally. The audio recordings were transcribed verbatim.

### Data analysis

We opted to use reflexive thematic analysis (TA) as our analytical framework, enabling us to contextualize our analysis for healthcare and uncover intricate and underlying patterns of meaning within the available data [26]. In particular, we chose reflexive TA because this study aimed at a deep and nuanced understanding of the data that captures the complexities of developing AI for healthcare without rigid preconceptions [27]. Two authors (L.A.O, M.R.) led the analysis, and all the co-authors supported the process. We carried out inductive and deductive thematic coding of the data, initially line-by-line, using descriptive or latent labels (software MAXQDA). L.A.O. and G.L. coded all the AI experts’ interviews with coding sessions supported by M.R., S.M., and D.S. The first two authors L.A.O and M.R. developed overarching themes reviewed and agreed upon by the entire research team later. After iterative analysis and reflections, the team created major themes illustrating the practical ethical concerns of developing AI for healthcare. For this publication, the authors present examples of data without identifying information.

The researchers' backgrounds informed the interpretation of the data and led to actively developed themes that focus on big ethical questions of who is benefiting from AI and why. In behavioral and political science, person-centrism is a widely acknowledged paradigm that helps to question and reflect on power structures and how these affect patients. Although our positionality has informed our analysis, the research group engaged in frequent discussions and included different academic backgrounds (philosophy, ethics, medicine, psychology) to prevent a single or superficial analysis.

## Results

We developed three themes presented through representative data extracts (de-identified). Given that AI in healthcare is a multidisciplinary area, most professionals found themselves at the intersection of two or more areas of experience; for example, eight participants were medical professionals with AI experience. The acronyms used aim to illustrate the main field of the expert: MEAI for medical experts with AI experience, BE for bioethicists, DH for digital health experts, LE for legal experts, PE for those experts working in policy analysis, and TE for technical experts either in data, AI techniques or AI product development. To improve readability, the authors removed filler sounds and double words from the data presented in this paper and the Supplementary information. The sample characteristics are described in Table 1.

**Table 1:** Summary of sample characteristics

Sample	AI experts (*n* = 41)
Geographical location of AI experts	Switzerland (*n* = 13) Germany (*n* = 8) United Kingdom (*n* = 8) United States of America (*n* = 5) Netherlands (*n* = 3) Belgium (*n* = 1) Canada (*n* = 1) Italy (*n* = 1) South Africa (*n* = 1)
Gender	13 women, 28 men
Duration	Average duration approximately 40 min (30 min to 1 h)
Background (can add to more than 100% because of multiple expertise)	Medical (*n* = 13) Ethics or bioethics (*n* = 12) Technical (*n* = 9) Law (*n* = 6) Digital health (*n* = 5) Policy and economics (*n* = 2)

### Creating AI with an ethical purpose

This theme explores the main challenges of creating AI for healthcare with a purposeful perspective. Several AI experts questioned the reasons behind AI’s development and whether the justifications are enough to deploy it for clinical care ethically. In their words, some experts fear that AI is a “shiny new object” mainly developed to answer the desire for innovation rather than providing actual improvements. Some experts stated that the potential lack of purposeful development may lead to an overestimation of the theoretical benefits of AI while having limited practical application. Viewed this way, a clear purpose becomes vital to creating a useful, ethical AI that answers healthcare needs.

#### Resisting technology-driven innovation

Some experts challenged the notion that innovation is inherently positive. These experts expressed the (ethical) importance of justifying innovative products beyond their disruptive capabilities. They emphasized avoiding the temptation of treating innovative AI as a panacea capable of solving every healthcare problem (Table 2).

**Table 2:** Data extracts for sub-theme: Resisting technology-driven innovation

Data extracts for sub-theme
*I think there's a lot of naivety about what it [AI] can actually do, and I think it has a lot to do with a bit in an even understanding what the real problems are in healthcare and what to solve them (…). And it's often not really problem-driven but it's technology driven. So, they basically won't just for, to do something with AI, and maybe it's scrambled to get some good use cases where they can implement it. Instead of asking really what are the problems of physicians?, and can we solve them? And if, yes, if is it, does AI play a role? So, that, I think, the approach is often just, we want to use AI and then, just give us problems, we won't, we can solve everything...* ***[Rn29 (MEAI)]***
*Is it in every case an assistance the AI system can give? or is it also a disturbance? Uh, I think it's, now they, some, many people look at the improvements of the systems and "oh we should make it more transparent and so on", and, but I think some, more a misleading discussion, or a wrong place of discussion. It's also a technology drive discussion "yeah we can fix this with this another system that explains working of the other system" (laughs). So, it is a typical technology fix of a technology caused problems. And I think one should go in other way and decide what do we need to improve in the doctor-patient relationship and what can we let in this relationship and not look at "oh this data manipulating technology would lead to better results or so"*. ***[Rn11 (PE)]***

Some experts described how defining which problem to solve is a significant hurdle to creating an AI that is useful for healthcare. Experts described that when AI is not designed to solve a specific problem, it can become a hurdle, distraction, or simply ineffective for the application. In their views, AI design should be proactive, focusing on the intention to solve real healthcare problems and not reactive to what technology is capable of doing. One participant [Rn40 (TE)] mentioned the concept of “repairing innovation” and how designing AIs in practice is not about developing a new solution but rather requires adapting AI’s design to the context of the specific application and the (un)expected challenges.

#### Moving beyond theoretical usability

Several experts highlighted the gap between theoretical and practical objectives. While many AI publications and products have performed well in controlled environments (and in theory), there is a disconnect with clinical practice. There are questions about whether these theoretical results will translate into positive changes “in the wild”. Some experts worried that AI would become “cool” theories with optimistic results that fail to be implemented in the hospital setting because implementation is not the objective or that the results are not transferrable to real-life conditions. A few participants felt concerned about the relative emphasis on publishing AI results that seem good in theory but do not consider whether they can improve patient outcomes. The experts brought attention to the complexity of implementing AI solutions in healthcare and the importance of moving from research and development to actual deployment (Table 3).

**Table 3:** Data extracts for sub-theme: Moving from theoretical usability

Data extracts for sub-theme
*So I think AI is a bit of a buzzword that is associated with market growth and jobs and us doing things better and quicker. But doing things quicker doesn't mean better, necessarily. And, so I think that a sensible pragmatic approach would be to be skeptical and to allow long periods of piloting and trials until we have a full confidence that something really does work the way it is supposed to work, rather than jumping to conclusions because, is, that something is able to detect something* ***[Rn14 (BE)]***
*There are lots of papers about what is the best model, let's say to predict a certain disease and then these models on, the papers are published, you know, in respectable machine learning journals. So, the peer review seems to be working in that sense, that nobody is, is faking a new model. (…) The question then becomes, how would you actually implement it in the clinic and not only that. How do you actually implement it in a way that is useful? So, I mean if the model basically looks at all the data and then says, "oh well, based on, on everything that I have seen, I predict that the patient, you know, will die in the next week". That probably is not very useful, you would like to know beforehand, you know, when the condition starts deteriorating. ****[Rn32 (TE)]***
*So obviously, the example, like everyone else to give, it's an AUC [area under the curve*^a^*] of 0.98 is, like sounds amazing, but if the 0.02 is everyone in your data set who is Black and everyone else in the 0.98 is everyone who is white like that's a problem, but the AUC looks really good. ****[Rn39 (TE)]***

^a^*Technique to measure the performance of an AI*

### Balancing AI for different healthcare stakeholders

While the first theme focused on AI as an object of development, this theme explores those who shape and benefit from AI solutions. Some experts were concerned about who benefits from AI and whether AI solutions respond to the needs of patients and doctors. Some experts mentioned the tensions between optimizing processes, increasing profits, and maximizing patients’ benefits. In experts’ opinions, the question of who should decide on the (ethical) acceptability of AI’s development remains open and requires public discussion.

#### Considering stakeholders’ requirements

AI experts questioned whether AI focuses on the needs of those impacted by its usage. Regarding patient care, a few experts expressed how AI may not be genuinely patient-centric as patients’ views may be systematically omitted from AI’s development (Table 4).

**Table 4:** Data extracts for sub-theme: Considering stakeholders’ requirements

Data extracts for sub-theme
*Does this AI system really have your interest at heart? And, I think it's also sort of potentially concerning in all of those areas of healthcare where patients have quite legitimate views on which of different set of possible treatments they would want. Because I think it is a risk. So, that the AI system says that this is the best treatment (…). Yeah, but you could imagine AI advice being converted into slow pressure to take the best treatment, unfortunately. And sort of the fact that in many areas the best treatment is the one of the possible reasonable treatments that I want being sort of overlooked...* ***[Rn5 (BE)]***
I*'m skeptical about rushing to use something that is automated just because we can. Just because we can, doesn't mean that we should(…). So, what's important for me is not that we have a product that works, but that product that reduces health inequalities that is able to work for the most disadvantaged people within the society, for those who typically struggle to access services in the first place...* ***[Rn14 (DH)]***
*(…) Where we are sort of asking the doctors, like, uhm, "Okay, you try this out, like, from your perspective, like, what is the problem that you want to be solved?" Maybe we're not solving the problem that you want solved. We're solving the problem we wanna solve. So, like, what are the problems from your perspective? Like, how can we address some of those, you know. What, what would the system look like if it was useful for you. How would it fit in to your workflow?* ***[Rn27 (BE)]***
*And in my eyes that has to really come from the clinicians that inputs, they have to come with problems and then people have to try to solve them and often, it's a bit the other way around it, that people with the solution, trying to find problems that they can solve. And that often leads to a lot of efforts and in the end, products that no one wants to use*. ***[Rn29 (MEAI)]***

A participant [Rn41 (MEAI)] described how AI’s development might be marketing-driven rather than oriented toward patients’ needs. A few experts brought bioethical principles of justice and fairness into the discussion and how important it is to consider the distribution of benefits for patients and doctors. A lingering question is whether AI solves the challenges patients and doctors face, or if it focuses on the goals of the technology industry.

#### Tensions between incentives

Following the above questions, some experts described the tensions between benefiting those in healthcare and those working for the industry. In contrast to healthcare, where patient benefit is an essential incentive of care provision, those developing AI may be interested in profits or operational efficiencies. A few experts voiced concerns about the entities or people responsible for setting AI standards and pleaded for the critical examination of AI’s adequacy for healthcare requirements (Table 5).

**Table 5:** Data extracts for sub-theme: Tensions between incentives

Data extracts for sub-theme
*Because private companies have different motives than doctors, and the protocols that hospitals have around diagnosis and treatment are designed not with the incentives that a private company has, but with incentives of like patients' health and like international standards and things like this. So, I think that these things are in conflict with each other*. ***[Rn4 (BE)]***
*The medicine sect[or] is used to, that the community decides about the appropriateness of statistical methods and about, degrees of, statistical features, significance and reliability, and so on. And these are standards, discussed and negotiated in the community itself and this approach, I think, you have to also, to shift it or, pull it on the AI systems. That the medical community decides about the standards, what is reliable, what is robust, what is appropriate for this, economically feasible. It's also a question, and not, I think with the whole digitization, the people, feel like that it, that the systems came from heaven, and we have to accept all, and we have to trust, it, the providers and the developers, and I think it’s a falsery. I think the medical community has to decide about such crucial questions, it's about the trade of economic and medical benefits, and, it's, it's about the distribution of risk. And, this should not be left to the developers and the provider*. ***[Rn11 (PE)]***
*With the Internet of medical things. And, then this is just another way for hospitals to make money, for companies to make money, for doctors to make money, and to just, you know, do things faster, to compromise. You have to convince me that this is the best thing in my interest as a patient. And, I don't think we're doing a very good job of that*. ***[Rn24 (LE)]***
*You, everyone loves an AI standard, as you know. And then you end up like battle of the standards, and you have like 15,000 standards. And, so, I would be interested, like who gets to select and sign what standard and why? And tied to that is, because standards get defined, tends to be an organizational, regional or national, sometimes super-national level, but group, the values that get baked into those things are very interesting. So, for example, do you be looking to minimize error for everyone a small amount? Are you looking to maximize benefit for a small group narrowly? Who loses out when you do those things? Like the values that they take into some of those standards...* ***[Rn39 (TE)]***

### Context-sensitive AI development

This theme explores the contextual factors shaping AI within the unique healthcare landscape. Some experts expressed how compared to other industries, healthcare is unique in that risk is high and health is fundamental. A few experts highlighted the importance of considering how established rules and standards govern healthcare. A notable concern voiced by a few experts was the apparent lack of awareness regarding ethical healthcare practices. In some experts’ views, these considerations would help dictate what is expected and ethically acceptable for AI’s development and implementation.

#### Healthcare is unique

Some experts explicitly expressed how healthcare is a unique context that cannot be compared, regulated, or guided like other industries. In their view, healthcare needs higher standards for AI development and implementation than, for example, retail or autonomous driving. Some experts mentioned that common product development practices, such as time-to-market, testing, and quality assurance standards, may need to be re-considered in healthcare. For example, a participant [Rn25] mentioned that testing a solution during AI product development is not simply a question of iteration as in other industries, because AI may bring unexpected risks and challenges in healthcare. In that sense, a few experts mentioned the importance of including a system perspective during the development of AI and the importance of considering the unique relationships and context dynamics of healthcare (Table 6).

**Table 6:** Data extracts for sub-theme: healthcare is unique

Data extracts for sub-theme
*And I think in healthcare is a one level higher concerning. So, what level of security you want to have? Because you don't, you say “okay, if the autonomous driver in a taxi is stopping at the wrong place, or whatever. It's annoying but I don't care. If it makes an accident, that would be bad but if I'm protected, okay”. But if I go to healthcare professional, and I have a disease and I have a health problem. I will, I'm looking for the maximum security that this, that they, that I'm recommended the right therapy, or I got the right consultation and this is what people do all the time. Because this is where they go to one physician, and they don't go to another, or they fly around the world to go to the right surgery blah-blah-blah. So, I think, we thought a lot about how, in which position would we see AI in the treatment concept for patients...* ***[Rn1 (MEAI)]***
*You have to think very carefully about the used context of the particular AI system you are implementing and how it's going to fit institutionally. How it's going to affect workflows and so on. Because we have already talked about, a lot of the ethical issues, sort of, there are risks that could happen if you implement it. So, even a good AI system could be implemented in ways which means that it creates problems in the healthcare professional-patient relationship, or other problems*. ***[Rn5 (BE)]***
*Healthcare is people’s health outcomes, it’s not just something you can iterate, test, and count on that it is something that people want. Even if people want this thing, it does not mean that this is what they should get, right? So, the role of experts in healthcare is clear that, it can’t go without it, because we can see what can happen with marketing especially, right? Ways that you think a product can save your live or whatever, and if it is not actually doing what is told. We live in a world of marketing and narratives. And so, I think in healthcare it’s really important that those things are accurate*. ***[Rn25 (DH)]***
*If we keep stating that the healthcare is, it's different to retail, for example, then different type of decisions or to guide the way that we engage private companies and the framework in which these private companies are operating. So we cannot have the same framework for data use for the development of AI algorithms to, you know, to use in retail and the same type of AI, like regulations for AI tools to be used in cancer diagnosis. So, these are very two, very different activities. Even if from the computational problem, they might seem very similar, in terms of the context in which they operate and, you know, and the aim is, it's a thing, I would say are fundamentally different. ****[Rn36 (BE)]***

#### No need to "reinvent the wheel"

Some experts pointed out the importance of considering the rules, standards of practice, and ethical codes that dictate what is ethically acceptable in healthcare. In their view, AI is not necessarily a new technique or ethical challenge, and many existing ethical frameworks could be initially applied for its development. A few experts noted how an awareness of ethical healthcare practices could be a solid foundation to guide AI’s development instead of creating new protocols that may be misguidedly technology-focused (Table 7).

**Table 7:** Data extracts for sub-theme: no need to reinvent the wheel

Data extracts for sub-theme
*I also don't agree with some who say that well this [AI] is a new technique, this is a new development, it needs new regulations. I disagree. If you look at it from an abstract point of view it's nothing different, it's what has been around for forever*. ***[Rn7 (LE)]***
*So, I mean, I think it's, in general, we have to apply the very same frameworks that we did in the past for other new technologies, right? I mean, if we think of CRISPR and CRASPR methodologies of genome editing or so, then we, I think, we also have to ask ourselves, "ok is this now something new?" I mean, we were able to do over expressing genomes in organisms or model organisms for a long time. And what, what kind of rules did we apply here? What was, you know, the ethical standards that we should, or recommendations that we should comply with? And I think, the same is true also now for these new technologies...* ***[Rn8 (TE)]***
*As a philosopher, sometimes I'm asking what are we doing? So, why do we so easily commit ourselves to a plan such as AI ethics? So, this is a bit scary. Because this is exactly what ethics should not be about. To say “well it is”. […] So, when we talk about medicine, we have to use the principles and standards of medical ethics. So, it's a completely crazy and stupid idea to say: “Well, we start from scratch, and now we think about AI ethics”. This is a kind of techno-specific focus, which is not really a good idea and which will cause concrete harm. ****[Rn9 (BE)]***
*I mean, you don't have to reinvent the wheel. There's lots and lots of folks that are looking into this [AI] now and have come up with various layers of standards and responsible AI use. So, at the very start, just pick the ones you like and start somewhere. You can fix them later, but you need to start somewhere. It's not going do you any good, like every ethics principle if you don't abide by them. So, what are the consequences if you don't ? ****[Rn24 (MEAI)]***

## Discussion

This research paper explores the development of AI and the considerations perceived by experts as essential for ensuring that AI aligns with ethical practices within healthcare. The experts underlined the ethical significance of introducing AI with a clear and purposeful objective. Experts expressed that beyond being innovative, AI needs to be meaningful for healthcare in practical ways. During the interviews, experts illustrated the ethical complexity of navigating the tension between profit and healthcare benefits as well as the importance of prioritizing the interests of healthcare professionals, and patients who are the stakeholders most affected by AI’s implementation. Experts highlighted the importance of understanding the context, the intrinsic dynamics, and the underlying theoretical foundation of healthcare during the development of AI. The three themes collectively call to deliver AI that serves the interests of doctors and patients and aligns with the intricate and context-specific healthcare landscape. For this to be achieved, those developing AI applications need to be sufficiently aware of clinical and patient interests, and this information transfer to the developers must be prioritized.

To our knowledge, limited evidence exists regarding the practical aspects of developing ethical AI for healthcare. However, in a roundtable discussion by experts, the ideal future agenda for AI and ethics included the questions: “(i) who designs what for whom, and why? (ii) how do we empower the users of AI systems? (iii) how do we go beyond focusing on technological issues for societal problems?” [28]. Our results validate how integral these questions are within a specific context of application, namely healthcare, and how they can help recognize ethical pitfalls in AI’s development. Our results focus on readily understandable ethical questions such as: Is AI developed for the right reasons? And, is the solution benefiting the right stakeholder? These practical questions can help evaluate the ethical implications of AI in a more understandable and relatable manner [29, 30].

One participant mentioned the concept of “repairing innovation” originating from Madeleine Clare Elish and Elizabeth Anne Watkins. This concept adequately summarizes the challenges described by our experts of developing AI solutions in healthcare. Elish and Watkins stated that there is a critical role in examining and understanding how effective clinical AI solutions must be considered part of complex sociotechnical systems in their development [31]. They advocate seeing AI beyond its potential (and often theoretical) possibilities but centrally investigate whether AI addresses existing problems, exploring how and in what ways AI is integrated into existing processes as well as how it disrupts them [31]. For them, to repair innovation is to set new practices and possibilities that address the often unexpected changes caused by AI’s disruption and integrate them into an existing professional context. Collectively, our findings suggest experts saw the need to change the way AI for healthcare is currently developed. They often called implicitly to repair the guidance, process, and incentives that help make AI align with ethical frameworks.

The World Health Organization guideline for AI ethics states that implementing ethical principles and human rights obligations into practice must be part of “every stage of a technology’s design, development, and deployment” [32]. In line with their statement, ethical AI (and AI ethics) cannot be solely involved in defining the ethical concepts or principles that must be part of AI, but must help guide its development. However, the current versions of AI ethics guidance have had limited effect in changing the practices or development of AI to make it more ethical [3, 15, 33]. Hallamaa and Kalliokoski (2022) raise the question: "What could serve as an approach that accounts for the nature of AI as an active element of complex sociotechnical systems?” [33]. While our results cannot offer an answer to this question; the insights of this study suggest that developing and implementing ethical AI is a complex, multifaceted, and multi-stakeholder process that cannot be removed from the context in which it will be used. In that sense, AI ethics for healthcare may need to become more practically minded and potentially include moral deliberations on AI's objectives, actors, and the specific healthcare context. In this way, our study focuses on the practical ethical challenges that are a part of the puzzle regarding what “ought to be” ethical AI for healthcare. Further research is needed to answer which tools or methods for ethical guidance can achieve in practice better ethical alignment of AI for healthcare.

In particular, the experts in our study were concerned about the innovation-first approach. These concerns, however, are not unique to healthcare. While innovation may be positive when it answers to the specific needs of stakeholders and is context-sensitive, it can also be simply a new, but potentially, useless product. Although the RRI framework places great importance on creating innovative products that are ethically acceptable and socially desirable, there are currently no tools that can help determine whether an innovation fulfills the conditions for RRI [34]. RRI is mostly used to determine regulatory compliance, which means the assessment of whether an AI fulfills RRI may come “too late” when it can no longer be transformed to impact practice [11, 34]. Guidance to develop AI ethically and responsibly may need to shift to a proactive and operationally strategic approach for practical development instead of remaining prescriptive.

Within the frameworks that guide AI’s development, the question remains: Who is in charge or responsible for ethically aligning AI in healthcare? Empirical evidence suggests that development teams are often more concerned with the usefulness and viability of the product rather than its ethical aspects [35]. In part, these results are expected as software developers are not responsible for strategic decisions regarding how and why AI is developed [17]. While some academics have suggested embedding ethics into AI’s design by integrating ethicists in the development team [36], management (including product managers) may be a better entry point to ensure that AI is ethically developed from its initial ideation. In a survey, AI developers felt capable of designing pro-ethical AI, but the question remained whether they were responsible for these decisions [37]. These developers stressed that although they feel responsible, without senior leadership, their actionability is limited [37]. This hints at the possibility that operationalizing AI ethics may need to include business ethics and procedural approaches to business practices such as quality assurance [30].

For our experts, context awareness is undeniably important, and a systemic view of healthcare is essential to understanding how to achieve ethical AI. AI innovations by themselves do not change the interests that determine the way healthcare is delivered or re-engineer the incentives that support existing ways of working, and that is why “*simply adding AI to a fragmented system will not create sustainable change*” [38]. As suggested by Stahl, rethinking ecosystems to ensure processes and outcomes meet societal goals may be more fruitful than assigning individual responsibility, for example, to developers [9]. Empirical evidence collected on digital health stakeholders in Switzerland showed that start-up founders may lack awareness or resources to optimize solutions for societal impact or that their vision may be excessively focused on attaining a high valuation and selling the enterprise quickly [11]. Similar to our results, the participants in Switzerland reflected on the tension between key performance indicators focused on commercial success or maximization of societal goals [11]. It might be challenging to address this tension without creating regulatory frameworks for AI’s development and business practices.

In contrast to focusing on AI as product development, for example, ethics-by-design, Gerke suggested widening the perspective to design processes that can manage AI ethically, including considering systemic and human factors [39]. Attention may be needed to evaluate the interactions of AI with doctors and patients and whether it is usable and valuable for them. For example, an AI assisting diagnosis of diabetic retinopathy may not be helpful for ophthalmologists as they already have that expertise [6]. Along similar lines, digital health stakeholders in Switzerland described that due to the complexities in navigating the health system, innovators may lose sight of the “*priorities and realities of patients and healthcare practitioners*” [11]. Our results reflect these findings, showing that balancing AI for different stakeholders is challenging. Creating frameworks and regulations that change the incentives of AI’s development may be an opportunity to answer stakeholders' priorities and healthcare needs. For example, to encourage the development of effective and ethical AI applications, reimbursement regulations could incentivize those solutions that offer considerable patient benefit or financial rewards when efforts have been put into bias mitigation [40].

### Strengths and limitations

While research papers are abundant for theoretical discussions, there is limited empirical evidence on the practical challenges perceived by experts to develop AI for healthcare that is ethically aligned. Therefore, our results are important to provide evidence that may help bridge the gap between the theory and practice of AI ethics for healthcare. Given the thematic analysis methodology, we collected rich data and conducted an in-depth exploration of the views and insights of a wide variety of experts.

For the context of our interviews, AI is used as a general term that can lead to experts interpreting AI differently or focusing specifically on machine learning (and its black-box subtypes). However, consensus on the definition of AI remains elusive and a topic of academic and governmental discussion. While the European Commission has recently defined AI,^1^ the definition is still broad. They included any software that can decide based on data the best course of action to achieve a goal [41]. While we clarified the focus on supportive AI as CDSS during the interview, some experts brought different understandings of AI to the discussion, delineating scenarios where it would be more autonomous and unsupervised. This challenge is not exclusive to our research or to healthcare, but it reflects the fact that AI is an ever-evolving topic currently under conceptual and practical construction and where multiple open questions remain. Given that our research aims to be exploratory, identifying different interpretations of AI can be considered part of our results, and signals a broader challenge in which research and ethics guidelines may need to define and study AI as application-, subject-, and context-specific. While our study demonstrates how practical challenges during AI’s development may need ethical reflection, as qualitative research, our results cannot be generalized outside the study population, and more research is needed to explore whether similar insights can be obtained in other areas. For example, future quantitative research could investigate whether participants from different healthcare models (commodity vs social service) may have different views or fears regarding AI’s development for healthcare.

Moreover, the chosen recruitment strategy of a purposive sample may have introduced bias in the selection of participants, given the dominance of researchers who are men or come from high-income countries. While we actively invited participants from non-dominant backgrounds (women and researchers of the global south), only a few accepted participation. Therefore, our results widely represent the views of those in Western countries, emphasizing Europe. The subject of our study must be further researched in different technological, socio-economical, and international systems.

## Conclusions

This research paper explored the critical ethical considerations highlighted by experts for developing AI in healthcare. Our main findings suggest the importance of building AI with a clear purpose that aligns with the ethical frameworks of healthcare and the interests of doctors and patients. Beyond the allure of innovation, experts emphasized that ensuring AI genuinely benefits healthcare and its stakeholders is essential. The existing tensions between the incentives of commercial success or benefit demonstrated the importance of guiding the development of AI and its business practices. In that sense, experts considered context awareness vital to understanding the systemic implications of AI in healthcare. In contrast to a narrow product-focused approach, AI guidance may need a systemic perspective for ethical design. This study brings attention to these systemic practical ethical considerations (objectives, actors, and context) and the prominent role these have in shaping AI ethics for healthcare.

Developing practical solutions to the identified concerns may have a high impact. While there is yet to be an answer to addressing these challenges and further research is needed, our findings demonstrate the intricate interplay between AI, ethics, and healthcare as well as the multifaceted nature of the journey toward ethically sound AI.

### Supplementary Information

**Additional file 1.** Interview guideline.

**Additional file 2.** Additional data extracts per theme.

## Data Availability

All data extracts analyzed during this study are included in this published article (and its Supplementary materials). However, the complete datasets used during the current study cannot be made publicly available but can be shared by the corresponding author upon reasonable request.

## Abbreviations

AI: Artificial Intelligence
CDSS: Clinical Decision Support Systems
GDPR: General Data Protection Regulation in Europe
ML: Machine Learning
RRI: Responsible Research and Innovation
EKNZ: Ethics Committee for Northwestern and Central Switzerland
HRA: Human Research ACT of Switzerland
TA: Thematic Analysis

## Footnotes

1 “Software (and possibly also hardware) systems designed by humans that, given a complex goal, act in the physical or digital dimension by perceiving their environment through data acquisition, interpreting the collected structure or unstructured data, reasoning on the knowledge, or process the information, derived from this data and deciding the best actions(s) to take to achieve the given goal” [41].
