# Supplementary material for: Integrating ethics in AI development: a qualitative study
Source: BMC Med Ethics. 2024 Jan 23;25:10. doi: 10.1186/s12910-023-01000-0 (PMC10804710; doi:10.1186/s12910-023-01000-0)
Supplement: Supplementary file 1 — Additional file 1. Interview guideline. [file 12910_2023_1000_MOESM1_ESM.docx]

**Interview Questions**

1. **Introductory questions**

| **#** | **Questions** | **Follow-up probes** |
| --- | --- | --- |
| 1 | Can you tell me a little bit about yourself? | - What is your background? - Where are you located? - What is your role? - Can you tell me more about your current projects - Do you work with ML/AI? - How did you become interested in ML/AI? - What is your experience with ML? |

1. **General questions about using AI in clinical practice**

| **#** | **Questions** | **Follow-up probes** |
| --- | --- | --- |
| 2 | I would like to start discussing using AI/ML in clinical practice.  What do you think about using ML in healthcare? | - In which cases do you think is useful and NOT useful? - Has it been beneficial so far? |
| 3 | How/Where do you think ML can/should be implemented in clinical practice? | - Is this a high priority need in clinical practice?   - Is there any specific application that you think would be the most useful? |
| 4 | What do you think about using ML for supporting doctors and patients in clinical decisions? | - Do you have any concerns? - Do you see any benefits? - Could it affect   - Doctor-patient relationship?   - Trust?   - Autonomy?   - Informed consent? - Would understanding ML be relevant in this context?   - What type of knowledge? - How would you define explainability? |
| 5 | What would you consider the biggest challenges of using ML in healthcare ? | - What are the biggest obstacles to overcome? - What challenges have you faced using ML in clinical practice? - Do you have any ethical or regulatory concerns? - Do you think ML can cause concerns for physicians? - Do you think ML can raise concerns for patients? |
| 6 | What would you consider the biggest challenges of using ML for supporting doctor and patients in clinical decisions? | - How do you think policy makers should be involved? - How do you think clinicians should be involved? - How do you think computer scientists should be involved? - How do you think ethicists should be involved? - How do you think patients should be involved? |
| 7 | I would like to know how you think we could advance the implementation of ML for the analysis of health data? | - How do you think policy makers should be involved? - How do you think clinicians should be involved? - How do you think computer scientists should be involved? - How do you think ethicists should be involved? - How do you think patients should be involved? |
| 8 | Which regulatory aspects are important for the implementation of ML in healthcare? | - Which regulations do you think are important?   - Would you prefer a strong regulatory framework, where different usages and situations are defined and there is a quite clear procedure, or a soft regulatory framework where general usages are defined but the particular practice can still be rather discretional? - Do you know any regulatory frameworks that are applied? - Which aspects need a lot of consideration? - Are there any major concerns that should be addressed by regulations? |

1. **VIGNETTES**

**Cardiology cases (comparison if patients’ basal risk change answers - context related questions):**

**If the interviewee has mentioned another case. Ask them to expand on it and ask the same questions/probes related to their example.**

Let’s consider a fictional scenario where someone owns a smartwatch. This smartwatch uses artificial intelligence to check the functioning of the heart (like heart rate, respiration rate, saturation, …).

**Scenario 1:** Jane is 40 years, has no previous diseases, feels healthy.

**Scenario 2:** Max is 70 years, lives with hypertension and diabetes and feels healthy.

One day there is a pop-up message saying that they have a change in the rhythm of their heart (cardiac arrhythmia - atrial fibrillation) .

Additional medical detail (if needed):

*Jane has no family history of cardiac disease. Jane has no symptoms. Previous visit to the doctor all results were in normal standards.*

*Max takes medication for his hypertension and diabetes and has already some signs in previous visits of deteriorating renal function.*

*The smartwatch that they use has been validated to take ECG (single-lead) to diagnose atrial fibrillation.*

| 9 | How do you think these patients should react to the pop-up message? | - Do you think that they should believe the pop-up message? What are your thoughts on trust? - What criteria are important for you to trust the results? - Who should trust the results? - How important is this concept for you? Why? - What information or facts would you need to evaluate the suggested diagnosis? - Should Jane and Max visit the doctor? Emergency or request a normal consultation? |
| --- | --- | --- |
| 10 | If Max/Jane decides to book an appointment with the doctor, what do you think they should say to their doctor? | - How should they share the data of the smartwatch with the doctors? - Should they mention that the reason for consultation is the smartwatch pop-up message? - How do you think Max/Jane feel about using technology to provide them more information about their health and have the capacity to say this to their doctor? |

**Vignette to compare other case where the clinical decision is based only on information (comparison of positions between patients and doctors and if type of disease change answers- context related questions):**

Now we have a patient named Ruth, 67 years old and she is feeling dizzy. She decides to go to the clinic for a check-up appointment.

The hospital she visits is implementing ML technology and the doctor will be using it during the consultation to support the diagnosis. During the check-up, the doctor adds all the symptoms. The doctor mentions that there is a chance of diabetes and that she is at risk for complications.

Additional medical detail (if needed):

*During the medical interview, Ruth mentions the triad of diabetes (thirst, polyuria, and increased appetite). Ruth's blood sugar levels are high, also her haemoglobin A1C is high.*

| 11 | How do you think the doctor should communicate the usage of ML? | - Do you think that the doctor should mention the use of ML to support the diagnosis? - What should the doctor disclose regarding the usage of ML? - How should the doctor explain ML to Ruth? - What other information should the doctor ask, mention or share with Ruth? |
| --- | --- | --- |
| 12 | What do you think about Ruth’s consent to use ML? | - Do you think consent is necessary? When? - What do you think about using her data? Do you have any concerns about health data handling? - If the context of consent changes and the ML would be suggesting an invasive procedure (e.g. surgery to remove the appendices) would that change any of your previous answers? |
| 13 | How would you feel about the doctor using ML to support the diagnosis? | - Would it be necessary for the doctor to understand ML? To what degree? - Would it be necessary for the patient to understand ML? To what degree? - What does explainability mean to you? - How would you evaluate explainability? - How important is this concept for you? Why? - *Additional medical probes:*    - *How would you handle a disagreement between your clinical judgement and the machine’s suggestions?*   - *How do you feel if the context of this case will be in an emergency situation? Would that change any of your previous answers?*   - *How do you think ML should handle medical uncertainty?*   - *Would you feel supported by the usage of ML?* |
| 14 | Do you think using ML during the consultation will affect in any way Ruth’s relationship with her doctor? | - How do you think patients will react to the knowledge of doctors using technology to support their decisions? - What would be the advantages and disadvantages to the doctor-patient relationship of using ML during the consultation? - *Additional medical probes:*    - *How would you feel about telling patients you are receiving ML support to make clinical decisions?* |

**Vignette private-public relationship**

Let’s consider the two scenarios discussed and add that in both cases, the ML/AI used to analyse the data was a private company. For example, Apple would be the one analysing the data with the apple watch.

| 15 | What would be your opinion regarding the involvement of private companies? | - What do you think about sharing data from public hospitals with private companies? - Do you have any ethical or legal concerns regarding their involvement? |
| --- | --- | --- |

1. **Closing questions**

| 16 | Are there any other practical, medical, and ethical issues that you think are important for the scenario that we have not discussed? | - Is there some concern that you have that was not addressed? - Do you have any recommendations? |
| --- | --- | --- |
