# Supplementary material for: Integrating ethics in AI development: a qualitative study
Source: BMC Med Ethics. 2024 Jan 23;25:10. doi: 10.1186/s12910-023-01000-0 (PMC10804710; doi:10.1186/s12910-023-01000-0)
Supplement: Supplementary file 2 — Additional file 2. Additional data extracts per theme. [file 12910_2023_1000_MOESM2_ESM.docx]

# Theme 1: AI’s objectives: Creating AI with Ethical Purpose

## Sub-theme 1.1: Resisting technology-driven innovation

| Rn3 (BE) | So, I think there are a couple [hurdles], that have to do with the way that these algorithms are being developed. So, I think there are way more algorithms being developed than eventually applied in medical practice. And I think that's a huge waste, both of resources and of energy, of everything. And I think a lot of that has to do with that these algorithms being developed just by people who think: "Ah yeah, let's digitalize medicine and develop some fancy algorithm" without understanding how things really happen in in medical practice. And if you want physicians or nurses or patients to use such an algorithm you really have to understand what is going on in clinical practice. So, I think there's a lot of waste going on. |
| --- | --- |
| Rn6 (BE) | Yes, of course. And it's part of the hype. So we're part of the huge money machine going to that, and distracting from the problem of sustainable water and other issues. Really, I said that again and again, because I really think that it's a problem. It's just the 21st century is not the century of data, it's the century of water. And it's so, for me, it's really distracting us from the real problems of this world. And artificial intelligence won't solve them. |
|  | And really think how much money do we want to put into that. Yeah, much more fairly than done right now. Because it is so much pushed form Apple and co. And big pharm also who do not like evidence based medicine because big pharma wants to have product there and it is much more easier to do not run a controlled trial but to run a machine learning thing and link it to consumer groups and think "whoa, we are innovative, we want personalisation" and the evidence is not there. |
| Rn11 (PE) | but I think the medical community should not be passive but uh, discuss in more active way. And they have, uh, the knowledge for this say, develop this knowledge about, uh, uh, for instance, statistical features, the rate of false positives, the rate of false negatives and so on, and what is acceptable in different treatments or different areas. And I think this approach or this attitude to the systems would be more adequate. And also a discussion, how far, uh, should we, uh, let computers in, in the treatment to the patient, how far can medical personnel, uh, yeah, uh, how far can they be disturbed in their original working on and tasks and, uh, responsibilities. Is it in every case an assistance the AI system can give? or is it also a disturbance? Uh, I think it's, now they, some, many people look at the improvements of the systems and "oh we should make it more transparent and so on", and, but I think some, more a misleading discussion, or a wrong place of discussion. It's also a technology drive discussion "yeah we can fix this with this another system that explains working of the other system" (laughs). So, it is a typical technology fix of a technology caused problems. And I think one should go in other way and decide what do we need to improve in the doctor-patient relationship and what can we let in in this relationship and not look at "oh this data manipulating technology would lead to better results or so".I think it's ok that people do this research (laughs) and think about it, but I think from the medical perspective or healthcare systems perspective it should be better to ask what should we let in the doctor-patients relationship. |
| Rn12 (LE) | First, um, how good these systems are? As simple as, as that. In many cases the trouble of these AI systems with their own aims, is that we pose the wrong questions to the AI systems. Well, it's seeming to be a stupid thing, but in many cases that's the game changer. So, we have technological issues. |
|  | And as I told you before in many cases it's, it's weird, but that for sure it happens that the problems do not change on the AI system as such, but in the way in which we use that system. As I told you before, it doesn't work because we pose the wrong question and, and so, um the first class of problems of course, concerns these specific facts. |
| Rn25 (DH) | So, I think there is definitely-definitely a role for that [using AI as CDSS], but it also has to be a well-defined problem and the value, also to the doctors and the health care professionals has to be clear and not uhm. |
|  | Of course, just because everyone is using something does not make it more viable or in the same way just everyone believes something makes that right or true or wrong. If there is value in a sense, that is the thing for valuable products. Um, that’s what I mentioned before. Like if you have a product that you create maybe it will create you, make money in a short, in the short term like Theranos, right? They, but in the long term, there is no value. It will disappear. The only way to go, is in the long term. In hindsight things that are better last longer. And that’s only if, you know we have the quote. Sorry what was it? Hindsight 2020, right? Looking back, you see it in perfect vision. And so, you only know afterwards what the value is, right? And so, yeah so, I think, that would be the best indicator later and you probably gonna have a lot of companies that gonna test digital health products and say: “no, this is actually not providing us the value which we thought it was”. With machine learning products also because, again, a lot of things are still in their early stages where we don’t know exactly where the value is in. Where is the cost saving? Which health outcomes are improved? |
| Rn28 (DH) | And it's an intellect innovation that people have to think not just disruptive innovation to be rapping up. What does disruptive versus intellect innovation means in AI? It's that, disruptive means "I want to do something big and something loud and something unique in the world, to make up my mark as a company" versus intellect innovation means "I'm building something for my end user in terms of health, so that they can have a healthy and long living life but I'm going to do it in a conscious way, the best way I can, which includes understanding the risk and impact, which includes I will make sure that the data bias is not there, and the cognitive bias is not there, all the possible biases can be addressed and so, that I can create a much more ethical AI that can be used, uh, for my, for the patients, uh, and help, bring health equality and so on". So, it is what we need from AI is, we need an intellect innovation of AI with the ethical by design and that is going to bring revenue for the people, they're only look for revenue (laughs) but also, but also it's going to bring, patients a better life. |
| Rn29 (MEAI) | Okay. So, basically I mean normally I think there's a perception of of this being a big game-change in medicine and people just think, well we just have to take the data and train it and you can solve a lot of problems. I think there's a lot of naivety about what it can actually do, and I think it has a lot to do with a bit in an even understanding what the real problems are in healthcare and what to solve them because I mean, just to give an example, uh, put in my when I, when I discuss with commercial software, when there is a, they just have these AI terms in their, in their bullet points and they kind of need to fix it in. And it's often not really problem-driven but it's technology driven. So, they basically won't just for, to do something with AI, and maybe it's scrambled to get some good use cases where they can implement it. Instead of asking really what are the problems of physicians, and can we solve them? And if, yes, if is it, does AI play a role? So, that, I think the approach is often just, we want to use AI and then, just give us problems, we won't, we can solve everything. So. |
| Rn31 (MEAI) | I think a lot of, of current medical applications take mature machine learning problem, you know, you've got the endless data set, and you want to say "okay well this is, this digit is a 6 but not 9 or a 7 and not a 1" which is a type of, of classification task and it, it, it takes those models and wants to look for medical problems to apply them to but actually I think to be useful, we need to, be dealing with the problems that clinicians struggle with, like "okay, I'm not, uh, I haven't seen a patient who looks like this before, I would like to follow the expertise of a system that is seen many more patients than me and can maybe say no, this isn't a normally and we need to do further investigation". I think that is where AI could be really helpful and it's not, it's not done so much. That's, that's partly a failure of the imagination of doctors and being able to ask for these sorts of questions, but also, uhm, a tendency for machine learning engineers to want to apply, uhm, paradigms that they recognize, to clinical problems. And it's not always the right paradigm. |
| Rn34 (MEAI) | I think it's also integrating in the current society, which is more and more digitizing, and, digitalizing, and in this aspect I think, it's totally normal that we are at least trying to develop these tools [AI]. But the question is, do we want to implement them? In the end, are there secure enough? And are they really helping me in clinical routine? Or there are just making me more work? |
| Rn35 (MEAI) | Yes. So, I mean, when I have a clinic and I want to, I need to be clear, this is also big challenge, I see in healthcare, that the healthcare providers for example, need to understand what's the problem they want to solve with AI first. So, at the better, they understand they want to improve diagnosis quality, they want to improve efficiency, times, speed. So, they need to define this and then they can better also understand what they will need. They need to understand what kind of patient population they have. So there's also a constraint. And what kind of geography they are in. But also what are the expectations from their patient population? What are the expectations from their doctors? So that, it is also picked up, it's useful for them, what is being developed or used there. |
| Rn36 (BE) | And the problem can be in the inappropriate use of AI, in the sense that, you know, it's not all questions are then reducible or can be answered through, kind of machine learning model or an algorithm. By the algorithm there might be other type of issues that, should not be reduced to this type of questions, to be resolved, this type of problems to be resolved. So that would be, I suppose some of the risks of kind of, you know, introducing AI to, into healthcare. As well as, things like, uhm, over, you know, over-promising like the hype around, around, you know, the technology at the moment that people think that, you know, everything, you know. It's like they have a hammer and everything is a nail. |
|  | the biggest challenge it's actually finding out when is appropriate use and when it's not an appropriate use (laugh) kind of answering this question, and trying to contain their enthusiasm to use the hammer for everything. |
| Rn39 (TE) | I think it demonstrating that actually works like a lot of AI doesn't work. It doesn't save lives. It doesn't save costs. It actually just adds much more burden onto a system that's already very stressed. Like any doctor will jump for a solution that saves some time, saves them energy.And, you know, a, more, see a decision maker save some money. And, I think a lot of the reason why this stuff doesn't get taken up is because the barrier to doing that is too high or actually thing don't really solve a problem. It's so clear that when some technologies, really fly , so, for example, this is not AI, It's just like some silly little gimmick thing but that a technology that's, that was, it absolutely exploded in the pandemic, from a company that basically added just a little toolbar in primary care physicians kind of bottom right-hand corner, which allowed them to interact with their patients in a much more kind of dynamic and easy way, where patients receive texts. And it, you know, it went gangbusters because, you know, clinicians found it really easy, saving time. It was, it enabled them to do their job. So, I don't think that it's a system not adopting it. I think it's about the people who build a solutions, don't really understand how the system works, don't understand incentives and in many cases are not actually solving a problem. Because a moment, a thing has real cost or life savings, people always adopt them. So, it's, it's not the system, it's the fact I think the solutions are crap in general. |
| Rn40 (TE) | So for example I read very study about sepsis, what it's from the Duke University, so they trained a sepsis detection algorithm and but it works locally and they also, uhm, describe the process, and they call it repairing innovation. So the process that was necessary to implement that algorithm. Uh because there's, you know, I really recommend you to read that study because they uncover topics that you don't think of when you are designing an algorithm, such as all of sudden the nurse is telling the doctor what to do, which is kind of a hierarchy change, right. Usually, the doctor tells the nurse what to do. So, this is something and they had to define strategies for how to overcome these challenges, yes. So that's why they called it repairing innovation so you have an innovation but then you need to repair it to really adopt it. And that I see it works locally, currently. So because it's so much effort and research teams, are local, and then they have the context, the nurses and so. So all that is needed so, a team is needed to really implement that. |
|  | I don't like the term AI anymore (laughs). We talk a lot about AI and somehow it became buzzword, uhm, I feel it's a bit like, we talk a lot about MP3, but we're not talking about music, what is really creating the value. So I think AI, so, it's just a technology, and it can be used for actually music, it can be used in healthcare, it can be used for, googling, or anything, yeah. So it's, what we need to focus on is really the application, yeah. So what, what do we want to improve with AI. Like the example you gave. So the, it's basically a pattern analysis, yeah, that has been around before. Uhm it's, I feel like it's has become a buzzword and we should not focus on, making policies for AI but really for the specific use-case, yeah. So I think it needs to be, uh, categorization of different use cases and you need to treat them differently, yeah. |

## Sub-theme 1.2: Moving beyond theoretical usability

| Rn6 (BE) | Of course, for sorting data and for finding patterns, I think for the main part, if we use machine learning in the same critical way as we use other technical means, and we do that in a good way, not enthusiastic, but in a critical way, knowing limits, thinking of ones, that we think of ourselves still. And that we know that they can be biases and wrongs and that we do not stop thinking. |
| --- | --- |
| Rn9 (BE) | And then you, but then you can see well when it's not about just saying the context is important, but each context has established institutional structures, has an established procedural pathways, which, of course, can be optimized. Where you can say: well, there are flaws within or, maybe, there are severe problems. But then you start to say: well, how can we develop a technology or how can we use this technology to optimize the concrete problems within this institutionally defined process? And I think this completely changes the debate. Because then you have to understand the processes but you have also a set of solutions and then the code of conduct for example, which is still in charge. So, it's not the case that when you use for example, um, an AI-driven support system in tumor board. So the doctors are still there. You can't say you replace them. But this is another debate. So, it will not automatically appear that, or come into place that all the doctors are away and you say: well, the patient is alone with this system. You can frame it this way but then it's, it's a question of responsibility and it is a decision you have taken on a political or on governance level. |
| Rn14 (BE) | And I don't even know if I fully understand what AI really means, even though I work in that, in that aspect. So I think AI is a bit of a buzzword that is associated with market growth and jobs and us doing things better and quicker. But doing things quicker doesn't mean better necessarily. And so I think that a sensible pragmatic approach would be to be skeptical and to allow long periods of piloting and trials until we have a full confidence that something really does work the way it is supposed to work, rather than jumping to conclusions because, is, that something is able to detect something. So, I think that, it's good if we exercise caution. If we have good-quality data. Good quality research, research that has been replicated by many different research groups, not only one, but many different research groups. And if they all come to the same conclusion, that there is a product that, that works and is safe and equitable and serves everyone, rather than increasing inequalities in society. Then that could be implemented. But again, under the scrutiny, monitoring, and further audit to make sure it works. So it could be a useful tool, automation could be a useful tool, but I think that like you said, things are happening very fast, perhaps too fast. Too fast to the point that we can really understand how things work and whether they work the way the should. So I would say, we really need to wait and have, and collect data, and have more data to then have the degree of confidence that we are doing things right. |
| Rn24 (MEAI) | So, the single biggest challenge to fixing problems around the world that have to do with healthcare, have to do with changing the behavior of healthcare professionals and patients. It's the single biggest challenge. I don't need to tell you or anybody else when was the last time you tried to change somebody's behavior. That's it. So, if you're thinking that just because we have shiny new objects people are going to use them. I'm sorry, but you're sorely mistaken. |
| Rn31 (TE) | This idea that you in the community it is much more easier to publish a paper that you can show reasonably well that it works on data to predict certain condition within the hospital setting. But then the actual step of implementing that in the hospital has lot more implications. The first one being that it then becomes an engineering project. So it's, you're not worried too much about the science once you, once you develop your model and you show that it works, then it becomes an engineering project because you have to go to the hospital, you have to embedded it in the existing systems, you probably have to change the way your predictive model works because it has to now receive online data all the time. It has to make decisions based on that. So that's, that's the first technical step that I see is very difficult to overcome. |
| Rn32 (TE) | there are lots of papers about what is the best model, let's say to predict a certain disease and then these models on, the papers are published you know, in respectable machine learning journals. So, the peer review seems to be working in that sense, that, that nobody is, is faking a new model. The gap is that between this model that was created, we know in with a publicly available data set or with a specific hospital data set. The question then becomes, how would you actually implement it in the clinic and, and not only that. How do you actually implement it in a way that is useful? So, I mean if the model basically looks at all the data and then says, "oh well, based on, on everything that I have seen, I predict that the patient, you know, will die in the next week". That probably is not very useful, you would like to know beforehand, you know, when the condition starts deteriorating. (...) I think that's one of the key aspects because in order for these models to work, they need to be closely, ahh, coupled with what the doctor does on the day-to-day basis. They, they need to be fine-tuned. |
|  | you know, I, I'm partly to blame, we don't know, we don't often look at these questions, you know. Our goal is, is sometimes minuscule. We want to see how accurate the model is, or how early can I predict this compared to another algorithm that somebody else proposed. But, these additional questions are not being asked. |
| Rn33 (MEAI) | The positive predictive value is extremely low [of an AI, for example]. So, if you imagine this and it's exactly what we observe with AI doc, implemented in Geneva in the emergency rooms in real patient for real radiography. Is that when the system tells you, there is a pulmonary embolism don't trust it. When the system tells you, there is no pulmonary embolism, that's fine. The positive predictive value is horrible. The problem is that you have 100 times more people, where the system tells you, might, there might be, uh, so if it's a mammography and breast cancer, it will be, uh, war crime to use these systems because you will have to do biopsies in millions of women in the world for nothing. And they will have stress, and they will change their life and other things. |
| Rn34 (MEAI) | I think another fact is also that you might develop something, pretty cool in research, but you're not implementing it, or you're not doing, how to say, a comprehensive pilot phase. So, you never get like a real field study, but you have a very cool tool and a theory. |
|  | Because I mean even if the tool is cool in theory, if it's only helping maybe 1 people out of 1 million, I'm not sure if it really helpful even though it would be helpful for this one person. And also the other factors, does it, or can it cause harm? So, yeah, you need to evaluate those precisely before using it. |
| Rn36 (BE) | But seems to me that someone is to be checking what gets released and to whom and when and how, rather than just allowing people to, you know, develop and you know some algorithm and then just with then to see how it performs and you know, and then kind of check if they're getting anything or not. So, that seems to me to be a kind of inappropriate way of introducing an AI supported tools, for, you know, in the healthcare space. |
| Rn37 (MEAI) | So for the supporting of the decision I think it can only be a good thing. I think you still have to think very carefully about, how to integrate it into the workflow so that it does not create more work for the clinician, I think that's very big topic and to be addressed. |
| Rn39 (TE) | So obviously, the example, like everyone else to give, it's an AUC of 0.98 is, like sounds amazing, but if the 0.02 is everyone in your data set who is black and everyone else in the 0.98 is everyone who is white like that's a problem, but the AUC looks really good. So, and that's a really trope example, but I am interested in values baked into, what does, what positive values should be prioritized not to AI systems. Uhm. There's probably the top of mind questions. I reckon. |
| Rn40 (TE) | See, I don't think the challenge is the development, so much, yeah. So there's, there are a lot of data sources, publically available data sets nowadays. So the problem I see, so, also with covid 19, I think it, it taught us the lessons that we had, there was a really nice article saying that there are thousands of AI algorithms now for covid 19. So for example helping to triage, no?. But none of them is actually used in clinical routine. Zero! So there have been thousands of research teams working on an AI algorithm, but none of them actually made it to the clinical routine, and for me that is a call for failure, really that we are spending a lot of time, you now with money from the society to develop something, and then we don't make it to bring it actually to life that it can benefit society, yeah. So the question is, and that's what I'm asking for the, the reasearch part is, do they really have the right KPIs (key performance indicators), yeah. Because when I talk to, uhm, and, and we are working together also with university, uh, with, yeah, AI teams, and, but in the university their goal is to publish an algorithm. Their goal is not to improve patients' outcomes, yeah. So when they stop is when they publish the algorithm, but they don't think about the adoption. And that's also already a problem what I have seen for the development of an algorithm because, uhm, if they're not thinking about the endgame, how to actually use this algorithm in clinical routine, they are, could go completely the wrong path. So they could use data that is not really available in clinical practice. They assume that all of the data is available at a certain time point, although we know from the workflow from the clinicians, that sometimes they don't have x y z lab values available because they don't test it. So the algorithm would need to be able to work also with, like, with missing data. And this is for example with some AI models, uhm, machine learn models, this is actually not possible. So they're not thinking about really I say they're not thinking about the adoption. I think it's a lot of ways to what is currently being credited in, in the scientific world, yeah. |

# Theme 2: AI’s Stakeholder: Balancing AI for Different Healthcare Stakeholders

## Sub-theme 2.1: Considering stakeholders’ requirements

| Rn2 (MEAI) | I think you would need to demonstrate the value to the patient. So, if you can say “listen guys we don't really understand always how it [AI] works but it's better than we are and we're going to use the thing”. That's very bluntly put but the way we have to go. If the average patient understands that he profits from something even if he doesn't understand it in every detail that's probably feasible. |
| --- | --- |
| Rn5 (BE) | Does this AI system really have your interest at heart? And, I think it's also sort of potentially concerning in all of those areas of healthcare where patients have quite legitimate views on which of different set of possible treatments they would want. Because I think it is a risk. So, that the AI system says that this is the best treatment. If it's an area where treatment choice is, and should, be preference sensitives, then it should continue to be so whether or not the AI system says that sort of my preference might be slightly suboptimal. Yeah, but you could imagine AI advice being converted into slow pressure to take the best treatment, unfortunately. And sort of the fact that in many areas the best treatment is the one of the possible reasonable treatments that I want being sort of overlooked. |
| Rn9 (BE) | Because I think on the one hand, it may be used as a misleading concept. And this would be the case when we do think that some scholars do take it that way and say well the technology, technology itself is disruptive. And I would say, I'm not quite sure about that. So, you can, so one classic example is always the one of making fire, yeah. So, this, it can be really a good thing to use the technology of making fire and, but, of course, there can be power asymmetries and, so, that only a specific person or set of, or group of persons, has the right to make fire and then others are not allowed to. And then this technology becomes a problematic one. Because of issues of justice or injustice, marginalization, discrimination. But these are all societal phenomena. And this is the reason why I would say: well, disruptive technology is a misleading concept. If we focus on, on a specific technology and try to regulate this technology. I think there's also a problem of, with regard to the current debate about the AI-act and then all this kind of stuff. But on the other hand it's, it's a really good concept. Because it is true that technology on the one hand enable us to shape life forms and do allow us to find solutions for social issues but also cause transformations on the social level |
| Rn14 (DH) | I'm skeptical about rushing to use something that is automated just because we can. Just because we can, doesn't mean that we should. So, for me as a researcher, I would like to see data in the first place where the data is about efficacy, about safety of using machine learning. But to go a step further about how equitable that machine learning is. So, what's important for me is not that we have a product that works, but that product that reduces health inequalities that is able to work for the most disadvantaged people within the society, for those who typically struggle to access services in the first place. |
|  | So, I think, that broadly speaking, it's not only the issue with machine learning per se, but also with the kind of, the whole concept of digital health that if we don't invite people from minority background, from various religions, from those of the lower socio-economic status who were not likely to have a very good education, those with lower health literacy and digital literacy who don't really have access to cutting edge technology. So, they can't, for example, they don't use mobile phones or smartphones, if we don't invite those people to help us develop AI. Then I think we are making a mistake there. Because then, it will serve, it would definitely serve some parts of the society, but I think, this will lead to broadening of health inequalities and will also increase the disparity in health between the poorest and those who are sort of most affluent.  So, so my attitude, the, the reason why I'm skeptical, is because very often, many start-ups or health companies, they present data on safety and efficacy of the machine learning , but very little is there about, fairness, whether it's non-discriminatory uhm AI, whether it's equitable, whether people from various minoritized backgrounds, and I mean it here, you know the ratio of men, female and those who don't identify as male or female, whether people of various ages were really included into the, you know, training of the algorithm, whether people of minoritized ethnic populations uhm those of minority, you know, sexual populations, same-sex people, people who might, you know, be bisexual or homosexual or any different sexual orientations. People of certain, you know, religions. We would very rarely ask about religion when we develop AI. |
|  | AI product that we develop really serves everyone rather than being tested on white population of certain age and certain gender. Because if we develop our AI using only that population as part of the training, then that will lead to broadening of health inequalities rather than narrowing them down. |
| Rn18 (MEAI) | Obviously we need to take, we need to integrate them [AI] in the best way possible, keeping in mind that our objective, our final objective is always the patient, and it is always taking care of patients. So, this is our objective. So, the final goal should always be, the patient. |
|  | Sometimes, for some, sometimes or some kind of, healthcare new application, informatics application, these are another, stakeholder that should be, involved. Not simple but essential if you want to, create these kind of involvement and empowerment of patients in this new field of informatics, informatics technologies applied to healthcare sector. |
| Rn25 (DH) | Yeah, well you are getting these tools it’s like iterating a test thing. And some of the users of these tools will be doctors and medical professionals, right? So, things have to be tested and trained with them in the exact same way. It is just a different user, actually. And so, they are a pretty primary stakeholder. And so, their needs, they of course, also have to be considered and not only. It needs to provide a value for them too. |
|  | I think the other point is what I was saying before. I said, it’s not always clear for who the value is for, right? But maybe, the value is different for the end-user or the payer, and the value is different for the healthcare system, right? |
| Rn27 (BE) | I know people are doing this, but I think, it seems impossible to do without, some kind of really robust two-way communication where we are sort of asking the doctors, like, uhm, "Okay, you try this out, like, from your perspective, like, what is the problem that you want to be solved?". Maybe we're not solving the problem that you want solved. We're solving the problem we wanna solve. So like, what are the problems from your perspective? Like, how can we address some of those, you know. What, what would the system look like if it was useful for you. How would it fit in to your, your workflow? And I think, of course, part of that is the explainability questions, which I assume we are going in. But I think that is not the full picture of why doctors don't use or trust these systems when they don't use or trust them. I think it's also how they aligned with the doctors' goals. |
| Rn29 (MEAI) | Well, I think, I mean, it's definitely a very powerful tool, right? It's it's I think, but it, it needs to be in a, you know, well-defined role and like with, and it needs to have it, it needs to answer good questions, right? And that is not, also in medical research is often like the questions are the largest thing to come up with, not, often also the solution, but the question is really the creative and difficult part. And in my eyes that has to really come from the clinicians that inputs, they have to come with problems and then people have to try to solve them and often, it's a bit the other way around it, that people with the solution, trying to find problems that they can solve. And that often leads, leads to a lot of efforts and in the end products that no one wants to use. And talk about problems. So, basically, I think the problem is that identification. One big problem is the problem of, the identification of problems to solve with AI and often people that I know from that sphere, they often just, just think, well, we, I mean, in the end what the AI does is predict things, right? And as a clinician, I think, predicting things it's for me, it was never that interesting, right? It's not that, it's not your need and this was also obvious in covid times, like people developed algorithms who could maybe predict positivity of, covid tests which in the end doesn't really help, because, in the end, everyone has a PCR anyway, because it's, it has been rolled out in such a big scale, and in the end, you would, when you have a test, you just do the test a not an algorithm as a doctor. And of course it can help with some triage but it doesn't really solve problems other that in the acute term or, also things like that seem like a very good idea like predicting mortality or ICU admission, in the end, normally you have, you, these algorithms are trained data that for patients that are already in hospital, and for them, if, me as a doctor and an algorithm tells me "well this patient has a 80% chance of being admitted to ICU in the coming 72 hours", well, what do I do with that? because you cannot admit someone just out of caution to the ICU because they're, they always have, have limited resources. So, basically, you, you admit them when he's bad. And, and when you suspect someone is bad, like, or only, like somebody is under surveillance in the hospitals, so you could argue, well I increase the surveillance of the patient that the nurses checking more, but, but these are just not the real problems I think that, that needs to be addressed. And a lot of these things also showed up in covid, for example. |
| Rn36 (BE) | So, I think that, it could, depending on, you know, depending on all the previous answers that the AI tool is used for the appropriate type of problem, in the appropriate way, it can definitely help things because it can give them one, one more kind important point of information that will guide decision-making both for the patient and the doctor. But if we start thinking using AI for everything or we think that, you know, that, you know, if we're deferring all our decisions to what that says, then, I think it will make the, you know, the healthcare space or the relationship much more, much poorer than, than before. |
| Rn38 (MEAI) | Well, I think that how the so that, where the recommendations come from and how they're, how they are built, so how are, the AI is built? Hey, I would, one thing that I would consider, perhaps a challenge would be, where, where do you start? So, there may be particular interests, be they from pharmaceutical companies or from, other organizations that focus on certain diseases to include certain things rather than other things or certain diseases first or certain modalities first. I think that that's kind of a discussion that should be had in the public sphere to see where AI may be utilized the best first, in a way that benefits as many people as possible. |
|  | But patients and patient organizations right now are the most important drivers of, the how data could be used in the exceptions that I'm that I'm talking about, in terms of rare disease patients. What are they willing to provide? What are they, what are they, looking for, looking to receive? What are their needs? I mean, most of what I'm saying comes from what I've heard from patient organizations and from patients. And, and that, and they, anybody who is in a situation where they, they have a lack of adequate information, and even the healthcare system isn't built to address their need. Then they obviously, they need to be absolutely very, very much involved from, from the very beginning in the discussions, qualitative discussions and, and so on, "what do you think about this? What, how would you want your data to be used?" and, and have a real discussion similar to the one that's going on with any kind of genetic testing, whether it's related to, inheritance of a certain diseases or genes that may predispose somebody to a certain condition. These are similar discussions. Always have to involve the patients and their, and the caregivers, families. Yeah absolutely. Their, their benefit, is the goal, so they have to be absolutely involved. |
| Rn41 (MEAI) | so, unless there is a clear additional benefit in making AIs very human-like, because maybe they help, you know, they help lonely people, who might interact with an AI that makes them less, feel less lonely, or so, and where the human-like design is a feature and not a surplus aspect, maybe then it's justified, but, we should really think about that at the level of designing, creating, developing this AI, my sense is that this is often not, not done in that sort of human-centred, human-oriented way, but rather, driven by considerations like, how can we make the devices less, yeah, agreeable and, so more marketing-driven, run, rather than really oriented towards human priorities and human needs. |

## Sub-theme 2.2: Tensions between actors and incentives

| Rn1 (MEAI) | or me this is something I want to see, that it's used with my patients all over the world and I would love to, to you know provide it at a minimum price [for AI werables] and, and of course, a company then would say “no-no-no-no that, We don’t do this to make the world a better place but we want to get the revenue on it for our investors or whatever” and, these are points at, at the moment where, where you talk about revenue and, and commercialization. Of course, is something that academics, it's, it's not that we don't see this at all. Because the money for research must come from somewhere but of course, this is not the primary goal and, so, these goals getting this research done and, or programming these kinds of algorithms. Of course, this is something which aligns perfectly and, The way to provide it to the health care system then this is something where it could really diverge tremendously. Because if you propose that we do this for free, well no company would have a reason to do so. Of course, well, that, that's, that's. They have other responsibilities than we have. |
| --- | --- |
| Rn4 (BE) | Well, I mean, I think if you're talking about like private companies, if they are the ones that are making these machine learning systems diagnostic tools. I mean, I think that that introduces a lot of challenges. Because private companies have different motives than doctors and the protocols that hospitals have around diagnosis and treatment are designed not with the incentives that a private company has but with incentives of like patients' health and like international standards and things like this. So, I think that these things are in conflict with each other. So, I don't really see how a private company could develop a diagnostic tool with those different motives unless it's just, yeah, completely open to audit and like the whole thing is completely open to audit at any time. |
| Rn6 (BE) | So, one challenge is, that a lot of money is going into machine learning, in my view. More and more. And it is triggered by big pharma, it is triggered by the IT industry, and that, this is my basic thing as a social epidemiologist, also that I know that the public health measures that really help the population lie in very different areas, right? Social injustice and stuff. And that we always struggle with the money given to research and implementation of these measures, public health measures, good quality nursing, good quality, GP practice, and really struggle there; good support for, for patients with incapabilities. That is one huge challenge. So the injustice in the research area, we call this a huge problem, a huge interest. And this is my, I think, one of the biggest challenges. Also, having an impact on how we deal with the problems of machine learning and the way, we discuss it. |
| Rn11 (PE) | Yeah, I think, uh, we know it from the safety, uh, area, there is no 100% safety because, uh, no one can afford 100%, uh, safety. And I think there will be also no 100% accuracy and someone has to made the decisions, how much accuracy is enough or not. And, I wanna, who is doing such decisions. And, I doubt it's, with the proposed AI act approach of the self-assessment, I wonder, or I, I criticize the providers are not the right actors who should do it such kind of decisions, what is accurate enough and what is not. And, we, I think, we need, maybe, we need sector-specific for the whole medical area or we need, uh, for a subsectors of medicine. For instance, what is accurate enough in the treatment of heart diseases, cardiovascular diseases, or, maybe there are different levels of, accuracy requirements for different subsectors. But, I am not able to decide it, because, because I don't have thought about this enough. But, I think these are the questions,who, we have to decide it. And you don't, I think you, one, we should not trust, the providers who said "this is enough" because it's not a question that they don't have the knowledge, but they have, somebody, has to do ethical or political judgments and this should be made not by the providers and not by the sanitizations organizations. I think it's better to, when there is a community of medicines that is like, uh, you can compare this with statistical analysis. The medicine sect[or] is used to that the community decides about the appropriateness of statistical methods and about, degrees of, statistical features, significance and reliability, and so on. And these are standards, discussed and negotiated in the community itself and this approach, I think, you have to also, to shift it or, pull it on the AI systems. That the medical community decides about the standards, what is reliable, what is robust, what is appropriate for this, economically feasible. It's also a question, and not, I think with the whole digitization, the people, feel like that it, that the systems came from heaven, and we have to accept all, and we have to, trust, it, the providers and the developers, and I, I think it’s a falsery. I think the medical community has to decide about such crucial questions, it's about the trade of economic and medical benefits, and, it's, it's about the distribution of risk. And, this should not be left to the developers and the provider. |
| Rn21 (LE) | I think a lot of innovations will be driven by commercial companies and we'll have a role in this. I think accessibilities of new technologies are important. I think that's, that there's, really, that's really crucial. I would love to say that every data should be publicly available and should be able to be accessed by all researchers but, of course, then you remove all incentives to build and invest money in new technology. So and that's the kind of attention and balance that you need to find between, those developments. Yeah. Is there a clear answer to that? No. I think uh what we should make sure is that if you have public investments in new technologies, I think we should make sure that data is being publicly available and that the benefits of those investments are going back to the public. And I think that is accessibility is there, potentially on a low access fee but in an accessible way. This does not mean that private incentives are not possible. |
|  | I think if you have those, if this is being generated by private then of course, yeah, it is harder to make that case, but I think it's, it's fair to be honest about the goal and, I think it becomes a bit unfair if, for example, you are working on public interest, motivating research participants, to motivate them to participate freely, on an altruistic reason to participate in data gathering, knowledge development, and then making a technology that is only accessible at a high price. And I think that's, that's becomes ethically problematic and that's something that many people are not aware of and that's really a difficult balance how to make. I mean a lot of the studies are, I mean, I'm not ,not very, in an expert in this, but I can imagine that a lot of vaccination studies, for example, where you could say people gotten the vaccine for free, but, but we know that in practice you have kind of monopolies on vaccines. And that a lot of people participate for low amount of, or no amount, not sure about the details of those studies, but you have a lot of financial benefits. And, and again here is this balance and, and finding the right balance is, is a big societal issue and, and, and debates. And I don't have the answer either but, but um that's a finding this balance is, is really crucial in that regard. (...) You know the discussions about the vaccines that are being made, where you have a monopoly, where you don't have the technology is not being given freely away so, that people that can produce that in other parts of the world. So, you have this kind of tension here as well. I mean, the topic I know best is for example, uhm, myriad genetics, I mean, has been a company that invests a lot BRAC testing. For a long time, they give away genetic variants so that people could around the world access them. In 2016 they just stopped doing this and they kept that. Why? Because they knew that they could use this knowledge after the patent expired. Because they knew that, yeah, this knowledge can create a better tool, a better technology that other people wouldn't have. So, they chose not to make their data available in the public domain. |
| Rn24 (MEAI) | With the Internet of medical things. And, then this is just another way for hospitals to make money, for companies to make money, for doctors to make money, and to, to just, you know, do things faster, to compromise. You have to, you have to convince me that this is the best thing in my interest as a patient. And, I don't think we're doing a very good job of that. |
|  | Um, and in most instances the number one thing that people are concerned with as a healthcare executive, is to keep the heat, light, and electricity on. How do we maintain our profit margins, and can we increase that using artificial, or maintain it? Whether it's revenue cycle management. Whether it's reducing operational cost or waste. How do we maintain our margins? Now, you may not like that and that's just the nature of the beast in the United States, but it's a business. So, how do you maintain that margin. |
| Rn31 (MEAI) | So, I think the, where a company seeks to gain financially and it's therefore motivated by financial return, there is greater risk of abuse of the trust that people put in their products to ensure that the product returns financially. And I think that in that case where it can be assume that the entity developing the AI is acting from sort of, from some sort of ethically or moral stand point, so, for example, clinicians have to follow an ethical code of conduct, you know, benevolence, non malevolence, justice, autonomy (...). Whereas companies do not have to follow that sort of ethical framework. They can't be called up in front of the general medical council for having developed something unethical and for that, the practical way of regulating that is trough hard law. If they want to develop something that pays off financially, there has to be a financial disincentive for doing that in an unethical way and that, to my mind is the purpose of law for any application. |
| Rn36 (BE) | And, uhm, yeah, and making, and also kind of challenging all these concepts around trust and distrust, and, and, reliance and, you know, like, understanding what, what, like whom do we, whom do we need to trust for what? Like, when is trust appropriate? When, when we need other type of things like, other type of relations that are not just trust based relationships like o, other type, like, when do we need just reliable systems rather than trustworthy systems? Uhm. You know, sometimes we just need, you know, kind of stronger reliance, like good, good regulations. So, then that the system becomes trustworthy. Uhm. And also to make sure that whoever makes decisions about all this kind of things are, are particularly, these decisions are made with all the appropriate stakeholders being part of the conversation as well. |
|  | But I think we need a bit more than that and we need a thing, we need stronger roles and we need, a good reliable system that regulates that space. Because at the moment it seems to be, like a lot of different companies and people can come in, like in, for example, in kind of mental health, anyone can develop, an app and kind of upload it on, you know, on Google store and you know, nobody, nobody checks all those things. And claim to kind of provide some kind of mental health, uhm, care support. So, it, it's still like kind of a bit of a wild west in some respects and but, and not in others. |
| Rn37 (MEAI) | I think that, uh, I think there's a danger when you give too much power to a few people who are running the software. Uhm, that can do lots of different things. That they close it to other providers, I think that there's a lot of work that needs to be done in integration. |
| Rn38 (MEAI) | I think that one, one discussion that is very important to have in my mind and it's always so long going, is the responsibility for data privacy versus the responsibility for helping to make a diagnosis, helping the patient helping the community. Uhm. And this is a certainly a difficult balance to achieve. However, I think traditionally so many people who are sponsoring this research, they may be, they may have other interests obviously, whether again, as it's a pharmaceutical company or whether it's a patient organization or, you know, even a health care system or an insurance company, they may have their own, they have their own interests. However, there is a question about the interest of the patient and the responsibility of the company or the insurance company, or the pharmaceutical company, or whatever. In my mind, there is a responsibility that a company has who has specific knowledge, is working on a specific area which is neglected, for example, in rare diseases or orphan diseases to provide one information, they have and use it to the benefit of the patient and to the benefit of the community as well. So limiting too much the ability of a company to initiate these kinds of projects, let's say using, oh, collaborating with a data provider to help diagnose patients, you know, there has to be I think in that case, a different type of consideration for what advantage the patients might gain, what benefit they might gain versus the risk that they might have for being identified because they happen to be the only patient in, Basel Land with this disease, right? So, it clearly, there's one that's a good argument, but the other argument is very, very strong. That this may be the only way that they will be diagnosed in time to receive any kind of therapy. So… |
| Rn39 (TE) | I'm always a bit of interest in like who defines standards, you everyone loves, everyone loves an AI standard, as you know. And then you end up like battle of the standards and you have like 15,000 standards. And so I would be interested like who gets a select and sign what standard and why? And tied to that is, because standards get defined tends to be an organizational regional or national, sometimes supernational level, but group, the values that get baked into those things are very interesting. So, for example, do you be looking to minimize error for everyone a small amount? Are you looking to maximize benefit for a small group narrowly? Who loses out when you do those things? Like the values that they take into some of those standards, I think are very interesting because a lot of AI is based on grouped data, and groups operate in very very interesting different ways and has a major repercussions on some. |
| Rn40 (TE) | I mean, the private companies, they are main interest is also to make money, no? And also, on the other hand, speaking of myself, for me working on a private company is also important to have a purpose, yeah. So that the company, I'm okay that my company wants to make money, but I want to work for this company because I, I see the purpose of improving patient's life. Yeah so that is why I want to work for that industry. So, somehow we need to find a balance, yeah, that not, yeah, keeping the purpose as a company and really go step-by-step. So what is really harming as is, I don't know, expectations from, from the management that, you know, within a certain time frame this can be solved. And there will be a certain revenue expectation, you know, within two years we will make that much revenue with this technology. That causes a lot of pressure in the organization and then people sometimes need to, you know, uhm, make decisions, uh, that go, uh, with all that pressure, yeah, to that compromise on quality, yeah. So that is something I think, and, the, the only one that can control this is the policy-maker, yeah, to make sure that this algorithm or the solution is being developed step by step. And not getting pressured by the industry. Not letting them, and, and not having, you know, no corruption. I mean, we have seen that now with, uh, a little bit, a lot of, yeah (...). Yeah, so that they know that this algorithm, and they keep the industry interest under control. Yeah, the management interest to maximize profits in short, uh, time. Keep it under control, yes. You will make revenue and it's a good motivation to earn money with this because it helps, and it helps the patients. And it creates value, so you should also be paid for it. But don't over-jump certain stage gates. |

# Theme 3: AI’s context: Context-senstive development

## Sub-theme 3.1: Healthcare is unique context

| Rn1 (MEAI) | And I think in health care, Health care, is a one level higher concerning, So, what level of security you want to have. Because you don't, you say okay, if the autonomous driver in a taxi is stopping at the wrong place, or whatever. It's annoying but I don't care. If it makes an accident, that would be bad but if I'm protected okay, but if I go to health care professional and I have a disease and I have a health problem. I will, I'm looking for the maximum security that this, that they, that I'm recommended the right therapy, or I got the right consultation, and, and this is what people do all the time. Because this is where they go to one physician, and they don't go together, or they fly around the world to go to the right surgery blah-blah-blah. So, I think, we thought a lot about how, in which, in which position would we, would we see AI in the treatment concept for patient. |
| --- | --- |
| Rn5 (BE) | Well, I think it could. I think it depends again on the context. It's much more likely to be in certain areas of medicine than in other areas of medicine. And it all also depends on, well, if we implement it in the right way, with the right kind of systems perspective, that sort of which takes account off all of these factors about how AI actually is going to work in this particular context. Then of course we can guard against all of these things. None of them are inevitable, the possibilities, but we could organize AI implementation so that the risks of the negative side effects are reduced. |
|  | I think the most important thing is to see this as a system issue. You have to think very carefully about the used context of the particular AI system you are implementing and how it's going to fit institutionally. How it's going to affect workflows and so on. Because we have already talked about, a lot of the ethical issues, sort of, there are risks that could happen if you implement it. So, even a good AI system could be implemented in ways which means that it creates problems in the health care professional-patient relationship, or other problems we've discussed. So, I think you need to have that systems perspective. It's going to be implemented in a very-very complex healthcare, is a very complex system and it's going to disturb that system in some way and then we have to try to figure out how to mitigate any potential negative effects of that. |
| Rn12 (LE) | However, it's not so easy to start using AI in hospitals and the like because it's not only a matter of abstract scientific knowledge and how good such systems are, but how you, um, rethink the organization of what doctors and nurses do. And, on the other hand, since you mentioned the patients, this is another relevant issue. Two years of pandemic has taught us that the rationality alone, um, doesn’t fix or solve all our problems. And that's the reason why, going back to my work with the (international organization) in matters of governance, a crucial role is played by engagement and even in some cases of forms of, let's call them, co-regulation or coordination and co-orientation among all different stakeholders. And, and, well, that's it. |
| Rn14 (DH) | But I think it's important because we need to understand culture. When we deal with health, it's not just that we look at symptoms but rather people of certain cultures might have different views on their health and how they access healthcare. And I would say, you know, that there are specific people with cultures that may have typical views about vaccines, as we know it in the context of Covid-19 vaccinations and if we don't understand those, then, then I think that we wouldn't not be having a full picture in regard to how AI works and how AI serves society. |
| Rn18 (MEAI) | Healthcare sector has its rules. We like it, we don't like it, (laughs) this is not the, not the question. You need, you need to know, um, what means healthcare sector today. |
| Rn25 (DH) | I think, Because you are working with people’s health, it makes it harder to do normal testing with people. In normal product development you are supposed to get Ideas and a hypothesis and you test them as quickly as possible, so you could iterate on your products. But if you make a mistake with people’s health, it’s not like if they get mushrooms on their pizza instead of peppers. And so, this is a challenging. The thing which allows other fields to move more quickly or faster, you can test with people faster and, Actually, that’s one of the premises of product development, that you are always testing and iterating. And that’s a little bit harder in healthcare, um, because of the nature of the field. |
|  | Healthcare is people’s health outcomes, it’s not just something you can iterate, test, and count on that it is something that people want. Even if people want this thing, it does not mean that this is what they should get, right? So, the role of experts in health care is clear that, it can’t go without it, because we can see what can happen with marketing especially, right? Ways that you think a product can save your live or whatever, um, and if it is not actually doing what is told, um. We live in a world of marketing and narratives. And so, I think in healthcare it’s really important that those things are accurate. |
| Rn29 (MEAI) | Ok, Nothing to most important things that I have. I have I said maybe to summarize I can say about I think it's really important to get to solve the right problems and which is often not done and to create frameworks. We're actually people. And I mean, where doctors are convinced of using the sources of information and I don't see a clear path of how that is done. And maybe another thing is also like actually incorporating these tools in your everyday life. I think just the ergonomics of it. It's also far off from being usable most instances because of it's just an application running on the side that you have to type data in. And so that is also big barrier thing to implementation that is that naturally plans in, with your diverse tools that. |
| Rn36 (BE) | I mean again, I will go back to what we're saying about how do we make, I mean, you know, how do we decide that this is the appropriate methodology for the problem that we have at hand? when we know which values we want to maximise and what will be again, the best, best way to go about maximising this type of in the healthcare context in general. Not just in one particular problem. To acknowledge that the benefits of AI, but as well, as its limitations, That it cannot always do everything much better that there are aspects of the healthcare, relationships, in the healthcare comes, that cannot be reduced to computational problems or a computational kind of mathematical problem that needs to be solved with these tools. |
|  | if we keep stating that the healthcare is, it's different to retail, for example, uhm, then, then different type of decisions or to guide the way that we engage private companies and the, the framework in which these private companies are operating. So we cannot have the same framework for data use for the development of AI algorithms to, to, you know, to, use in retail and the same type of, AI, like regulations for AI tools to be used in, in cancer diagnosis. So, these are very two, very different activities. Even if from the computational problem, they might seem very similar, uhm, in terms of the context in which they operate and, and, you know, and the aim is, it's a thing, I would say are fundamentally different. So, I think this is, this is like, I think where things are happening. You have a lot of companies who use to operate in one space now entering a very different space but you cannot have, but there is no kind of, uhm, regulation or understanding of this change between, you know, Amazon sending you, you know, kind of Amazon Prime about like when, when will be the best time to show you like ah, ah, an add of, of shoes that you know, that you might want to buy and kind of doing the same thing about kind of a health, you know, in the health context. |
| Rn37 (MEAI) | I think fundamentally they should know how the healthcare system, that make it very difficult to integrate any third party platform into an electronic health record, for example. And, and, and that, so not relates to AI. So just more generally. And that must change. Uhm, so, so yeah, we one hundred percent need to make a better job, uhm, of, of that integration piece. |
| Rn39 (TE) | I guess there are some cases where, and people who are involved in making decisions at a group level, for example, a hospital may not have been involved, that you miss the kind of macro-effect of this inclusion. So, for example, if you go back to the, if you can think of, or if we will figure to say, uhm, I also really compose that kind of example, ohh yeah, uhm, people who moved on to kind of face time and call their GPs during pandemic, obviously became a lot, in some space became a lot easier to access your primary care physician because you could call them on facetime or whatever.And we had the situation in UK whereby, uhm, it was kind of very easy to access this virtual online GP. Uhm. It required them to de-register from their current GP. So sitting this kind of virtual GP somewhere and, and then when they couldn't therefore get an appointment in a real physical GP, they then turned up at the hospital in accident and emergency. And that a sort of thing that as an inadverted doctor you might, you might not notice or, or take into account, so as a patient, you couldn't care less 'cause you just want to be seen but as a decision maker you probably could quite easily see "okay. Well, the additional traffic that the ease of access to your doctor over here is going to cause a huge bubbling effect in the, you know, hospital system over there" and those are sorts of things, I think the kind of big dramas in technology in AI and in implementation is where we're seeing, so we have these bubbling effect in areas that people haven't necessarily seen when they build a point solution. They didn't realize that, you know, they like feel like squeeze a balloon and like balloons over there. |

## Sub-theme 3.2: No need to start from scratch

| Rn6 (BE) | I have the feeling that the evidence-based guys also in WHO, and worldwide, and the AI guys, regarding journals, regarding issues, they do not interact with each other. The evidence-based guys when they read articles in Nature by a technology, they think "This is rubbish". And we even do not talk about it because it is so stupid. And these engineers publishing in Nature by a technology, in machine learning “bla bla bla, they have no clue about evidence-based medicine methods and epidemiological methods, and the problems that we see when we when put such a complex intervention to practice”. And I really think that we need, uhm, public health, we, I would love to see that the WHO is much more, is doing, policy that is really putting these experts together, also on the level of Nature, of journals like Nature. Really making forums of evidence-based guys, public health piece persons, and bio technology piece persons, promoting new knowledge. Those enthusiasts. And really discussing core issues much more intensely. Because we are publishing different. Really, it's really, absolutely, we have two debates going on, and they ignore each other in a way of just looking like, you know: "We know, we know what the innovation is" and the other say "we know what rubbish you do" and won't help. |
| --- | --- |
| Rn7 (LE) | And I also don't agree with some who say that well this [AI] is a new technique, this is a new development, it needs new regulations. I disagree. If you look at it from an abstract point of view it's nothing different, it's what has been around for forever. It's like someone who drives around with tractor and runs over a child and the tractor can kind of take over because the driver is drunk, but it's still his fault. So, I think we don't have that many difficulties assigning fault or responsibility even in a world where artificial intelligence will be much more used. I would claim that the basic rules of adjudication or responsibility will still apply. |
| Rn8 (TE) | I'm so happy that I do not have to develop the regulatory for everyone. We have problems enough on our data level, so I actually did not think about this a, a lot yet. So, I mean, I think it's, in general, we have to apply the very same frameworks that we did in the past for other new technologies, right? I mean, if we think of CRISPR and CRASPR methodologies of genome editing or so, then we, I think, we also have to ask ourselves, ok is this now something new? I mean, we were able to do over expressing genomes in organisms or model organisms for a long time. And what, what kind of rules did we apply here? What was, you know, the, the ethical standards that we should, or recommendations that we should comply with. And I think, the same is true also now for these new technologies. |
| Rn9 (BE) | So, as a philosopher, sometimes I'm asking what are we doing?. So, why do we so easily commit ourselves to a plan such as AI ethics? So, this is a bit scary. Because this is exactly what ethics should not be about. To say well it is. So, sometimes, for example, in the debate about the AI act or the UNESCO pay bond on artificial intelligence, there is this idea that you can't use ethics to solve political problems or that you can't use ethics to find solutions for technical problems. So, this is not the case. And on the other hand, there are concrete standards. So, when we talk about medicine, we have to use the principles and standards of medical ethics. So, it's a completely crazy and stupid idea to say: “well, we start from scratch, and now we think about AI ethics”. This is a kind of techno-specific focus, which is not really a good idea and which will cause concrete harm. Because there are longstanding debates about, um, for example, um, the, the clinical assets to say in what kind of procedures do we face which kind of responsibility, liability. What are the standards for explainability and so on and so on. So, now in AI ethics, everybody is saying: “wow, let's talk about explainability”, and it's a bit, yeah, of course, you can do this. But when it comes to the question of AI DSS, and this is my concrete answer, then it is important to say: “well, we have, first we have to look on the institutional practices. We have to look on the pathways of the decision-making in the clinic, and then we can ask how do they change when we start to embed on”, so, in which way and in which point of decision-making we do integrate a kind of of machine agent, or a kind of machine learning system. But it's not, it's not really a good idea to say “well let's talk about the standard of the machine learning system and then we would put it into the clinical decision-making process”. |
|  | well, there are long standing debate about the principles of medical ethics, for example. And it's a nightmare that, so I'm talking a bit directly, but yeah, I think this is what makes it interesting. So, it's, it's a bit of a nightmare that, for example, we have a longstanding debate about the principle of justice, and it's important for medical aspects. And, then for example, the high-level group on ethics just changed it and said: “well, we dont speak of justice anymore we do speak of trustworthy AI” and say: “well, now the principle is fairness”. And, as a philosopher you, you, you're standing there and say: well, wait, there's a difference between concepts of fairness and concepts of justice. And you can't, and, and it has longstanding implications if you just switch them and just say: “well, okay, instead of the principle of justice I now use the principle of fairness”. And I think this is an example why it's so important to, to say what we do have is established standards in medicine and this is your point of course, then you need the transdisciplinary expertise, but also in philosophy we do have standards for specific contexts and we have long-standing debates with how we can embed the standards. So, when we talk and think about explainability or liability, we do have established procedures, um, to guarantee, for example, liability, and it is, it is not really understandable that we then, that you, that, but now we have the new technology and we start from scratch with this debate. So, this is not, so, do you understand my point or? |
|  | But to make it really short, I think it is important to look on the specific institutional settings with regard to the concrete context. So, I think it is the, the whole mass this, this idea of making technology trustworthy itself, is that you do not focus on, on these standards, and this was my example with regard to medical ethics. For example, in the clinic we do have standards, we do have institutional settings, structures, which, of course, are not good themselves, but which has a longstanding history why they are the way they are. |
| Rn11 (PE) | Because, uh, I think that, there is a standard what I talked about, the standards of reliability, accuracy, what is acceptable and what not, I think that, that, the community should, should develop such, such standards, and they used to. In the past, uh, they had a lot of discussion about acceptability of medical, clinical studies, about, acceptable statistical, that are used, about, failures rates and reliabilities and so on. You know, p-values and so on (laughs). And, what is acceptable and everybody knows it and everybody can verify it, and everybody can challenge it, the use of statistical methods. I think it's important that the medical community, not every doctor, uh, I think some doctors will be, too much, too great task for them when they are but, in an appropriate part of the medical community who can challenge the methods behind such AI systems. |
| Rn18 (MEAI) | one of the most important things is using a, sort of ethnographic approach, in my opinion. You need to observe the, what physicians are doing right now in their field, and you need to understand which are their needs, which are their processes, which are their, which is their, their time, okay? And after this observation then you can try to work on that and understand, and try to understand if our new technologies, if you want, machine learning, can be used by them, can be beneficial for them, and then integrate them. But one of the most important thing is, uh, observe In the field what they are doing right now. And not try to imagine that: "wow, this is super useful we think that this, that this machine learning tool is super useful and, and we, I mean, we think about that in in our offices" and then: "okay, this is super useful" and going into clinics and sometimes this kind of tools doesn't work. |
| Rn24 (MEAI) | So, in my view there needs to be standards, there needs to be ways of measuring it, there needs to be a reporting system, and there should be a component of a hospital Ethics Committee that deals with AI ethics cases. Best case, you would discuss it before. Worst case, things go South. Now, you have a morbidity and mortality conference that discusses the inappropriate use of artificial intelligence in that patient, just like a complication of any kind. What went wrong? Why? What were the systems issues? Who's responsible? How do we prevent this from happening again? |
|  | I mean, you don't have to reinvent the wheel. There's lots and lots of folks that are looking into this now and have come up with various layers of standards and responsible AI use. So, at the very start, just pick the ones you like and start somewhere. You can fix them later, but you need to start somewhere. It's not going do you any good, like every ethics principle, if you don't abide by them. So, what are the consequences if you don't. So, if I'm a professional society. You know, every time you join a professional society, there's this little paragraph that says you have to abide by the rules, principles, and ethics. And, you know of blah-blah-blah. And… everybody just checks off the box and signs it, but there's no, there's no monitoring, enforcement, or consequences if you don't, short of committing a felony or doing something that is so egregious that you lose your license, but that's not going to happen in this. |
| Rn25 (DH) | And especially, because the health system was not build for, like there are so many also just things like in healthcare or in the health system which exist that are old models, right, based on wooly mammoths, right? Like they operate in old fashioned ways. And even though something could be a better way to operate there are a lot of switching costs that are related. And that’s why you see even for example, in the US. lots of like new health systems popping up, right? When you start a new health system, Again, you have some that, digital health builds right into them. And so, Um, I think we gonna see a lot of changes in a lot of different ways, opportunities like this and also changes in established healthcare. |
| Rn31 (MEA) | At the minute, it seems like there's a lot of regulation for something that hasn't even fully emerged yet and it's stopping potential benefits from coming through. But I understand why we're adopting an anti-risk strategy as well, because there are a lot of potential harms, but I think because of the hype around AI, is treated with a lot more suspicion than the statistical, you know, we've been using logistic regression in medicine for decades without this heavy regulation around it. And you could just imagine if logistic regression was so like heavily monitored, it stops a lot of [...] |
| Rn33 (MEAI) | I have every week people coming to me and saying, telling me that, they are a start-up [removed name], they have built the system which is absolutely more genial. They have tested it with the mimics that they set, which is open source and they want me to buy it, to use it with patients in the hospital, and they agree to do half of the price if I give them the data. And, I said "eh, vous avez fait des etudes? Les validé?, they don't, not even understand what I said, " C'est reproductible?" quelle, uh, how can you compare the data set you used to train it? Mimics. And the population we have in [Switzerland city], is it the same population? Is it two different population? "Vouz parlez de quoi?" they don't understand even what we speak about. It's shocking. And when you say "Ca va pas", they are so unhappy that they think you are against AI because you protect physician, right? I am not a clinician, I am an AI guy, my team is only doing AI. We try to do something which is helpful not something which is, uh, uh, "completement cinglé". |
| Rn39 (TE) | when I talk to my husband so I'm not and obviously, I'm not a clinician by background and he slightly jokes, but he says, you know "designers will come to me and say, so, we've done some user testing, and you've got these sorts of patients, and he's saying that being like, you've spent, you know, two months doing that design, user-design work, and I could just tell you that like that (clicks fingers) because I see hundreds of patients a day. So, that's a really good use of your time". And I wanna, you could, you could therefore say the clinicians have a very narrow view of the world and actually it's quite useful to have a, you know, people like designers to come to kind of strive to pull them out of there, to pull out their holes, but also the same time like the clinicians really know what's going on, in, in their sector and can answer. So, I think it's, I think it's fair to say that there needs to be a little bit more clinical, there should be as much clinical input as possible into all of these solutions because actually, you can't really learnt what it means to be in the clinical environment all the time. You really have to have those people together. But beyond that, and obviously you, you would try people with the technical skills but beyond that, I really think it kind of really depends on what problem that is being solved. |
